# Supplementary material for: Control of lysogeny and antiphage defense by a prophage-encoded kinase-phosphatase module
Source: Nat Commun. 2024 Aug 23;15:7244. doi: 10.1038/s41467-024-51617-x (PMC11341870; doi:10.1038/s41467-024-51617-x)
Supplement: Supplementary file 1 — Supplementary Information [file 41467_2024_51617_MOESM1_ESM.pdf]

Supplementary Information for

Control of lysogeny and antiphage defense by a prophage-encoded kinase-phosphatase module

Yunxue Guo[1,2,3#], Kaihao Tang[1,2#], Brandon Sit[4,5,6#], Jiayu Gu[1,3#], Ran Chen[1,2], Xinqi Shao[7], Shituan Lin[1,3], Zixian Huang[1,3], Zhaolong Nie[1], Jianzhong Lin[1,3], Xiaoxiao Liu[1,2], Weiquan Wang[1, 3], Xinyu Gao[1,3], Tianlang Liu[1,3], Fei Liu[7], Hongbo R. Luo[8], Matthew K. Waldor[4,5,9]*, Xiaoxue Wang[1,2,3]*

*Corresponding authors. Email: mwaldor@bwh.harvard.edu; xxwang@scsio.ac.cn

The PDF file includes:

Supplementary Figures

Supplementary Fig.1: Effects of Pf4 and Pf6 deletion on biofilm formation and host growth.

Supplementary Fig.2: Sequence comparisons and predicted structures of the components of MPAO1 KKP module.

Supplementary Fig.3: Expression dynamics of KKP_{MP} genes via qRT-PCR and quantification of Pf4 and Pf6 phages via qPCR.

Supplementary Fig.4: MvaU is phosphorylated at multiple sites and phosphorylation of S67 in MvaU inhibits Pf phage production.

Supplementary Fig.5: KKP_{MP} is a toxin/antitoxin system.

Supplementary Fig.6: PfkA interacts with PfkB and PfpC neutralizes PfkA/PfkB toxicity.

Supplementary Fig.7: Cell morphology of the overexpressed KKP_{MP} components.

Supplementary Fig.8: KKP_{MP} provides phage defense by reducing plaque sizes.

Supplementary Fig.9: Further characterization of KKP_{SW} and KKP_{EC039}.

Supplementary Fig.10: Evolved T4 phages escape KKP_{EC039} defense.

Supplementary Tables

Supplementary Table 1: List of the Pf4 and Pf6 prophage genes in MPAO1 and their genomic locations.

Supplementary Table 2: Presence of Pf4 and Pf6 loci in the selected *P. aeruginosa* strains as analyzed by BLASTN.

Supplementary Table 3: RNA-seq reads of each Pf4 and Pf6 genes in biofilm and planktonic MPAO1 cells.

Supplementary Table 4: List of putative PfkA kinase substrates identified by phosphoproteomics.

Supplementary Table 5: Mass spectrometry results of purified $PfkA_{MP}$-His during co-purification of $PfkA_{MP}$-His and Supplementary $PfkB_{MP}$.

Supplementary Table 6: Mass spectrometry results of the purified protein Flag-$PfkB_{MP}$ during co-purification of His-$PfkA_{MP}$ and Flag-$PfkB_{MP}$.

Supplementary Table7: Mass spectrometry results of the purified protein His-$PfkA_{MP}$(N) during co-purification of His-$PfkA_{MP}$(N) and $PfkB_{MP}$(N)-Flag.

Supplementary Table 8: Mass spectrometry results of the co-purified protein band with His-$PfkA_{MP}$(N).

Supplementary Table 9: Mass spectrometry results of the purified protein His-FHA during co-purification of His-FHA and CSD-Flag.

Supplementary Table 10: Mass spectrometry results of the co-purified protein band with His-PfkA$_{MP}$.

Supplementary Table 11: Mass spectrometry results of the co-purified protein band with His-PfpC$_{EC039}$.

Supplementary Table 12: Bacterial strains and plasmids used in this study.

Supplementary Table 13: Oligonucleotides used for gene knockout and DNA sequencing.

Supplementary References

Supplementary Figures

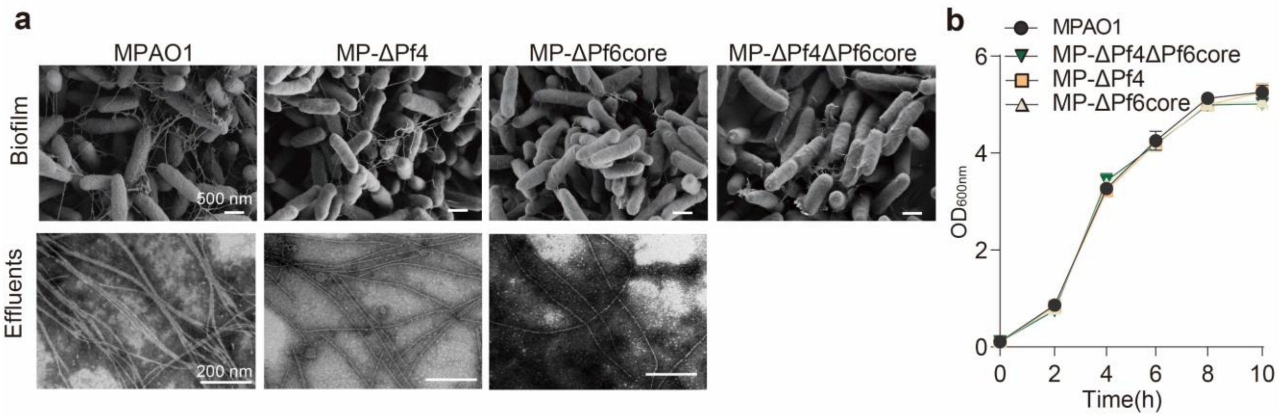

Supplementary Fig. 1 Effects of Pf4 and Pf6 deletion on biofilm formation and host growth.
a, Transmission electron microscopy (TEM) images of biofilms (top) formed by MPAO1, MP-ΔPf4, MP-ΔPf6core and MP-ΔPf4ΔPf6core on day 6, as well as biofilm effluents (bottom) collected from day 6 biofilms of MPAO1, MP-ΔPf4, and MP-ΔPf6core.
b, Growth analysis of MPAO1, MP-ΔPf4, MP-ΔPf6core and MP-ΔPf4ΔPf6core in LB medium at 37°C starting from an OD_{600} of 0.1. Three independent cultures were examined, and the data were presented as the mean ± SD.

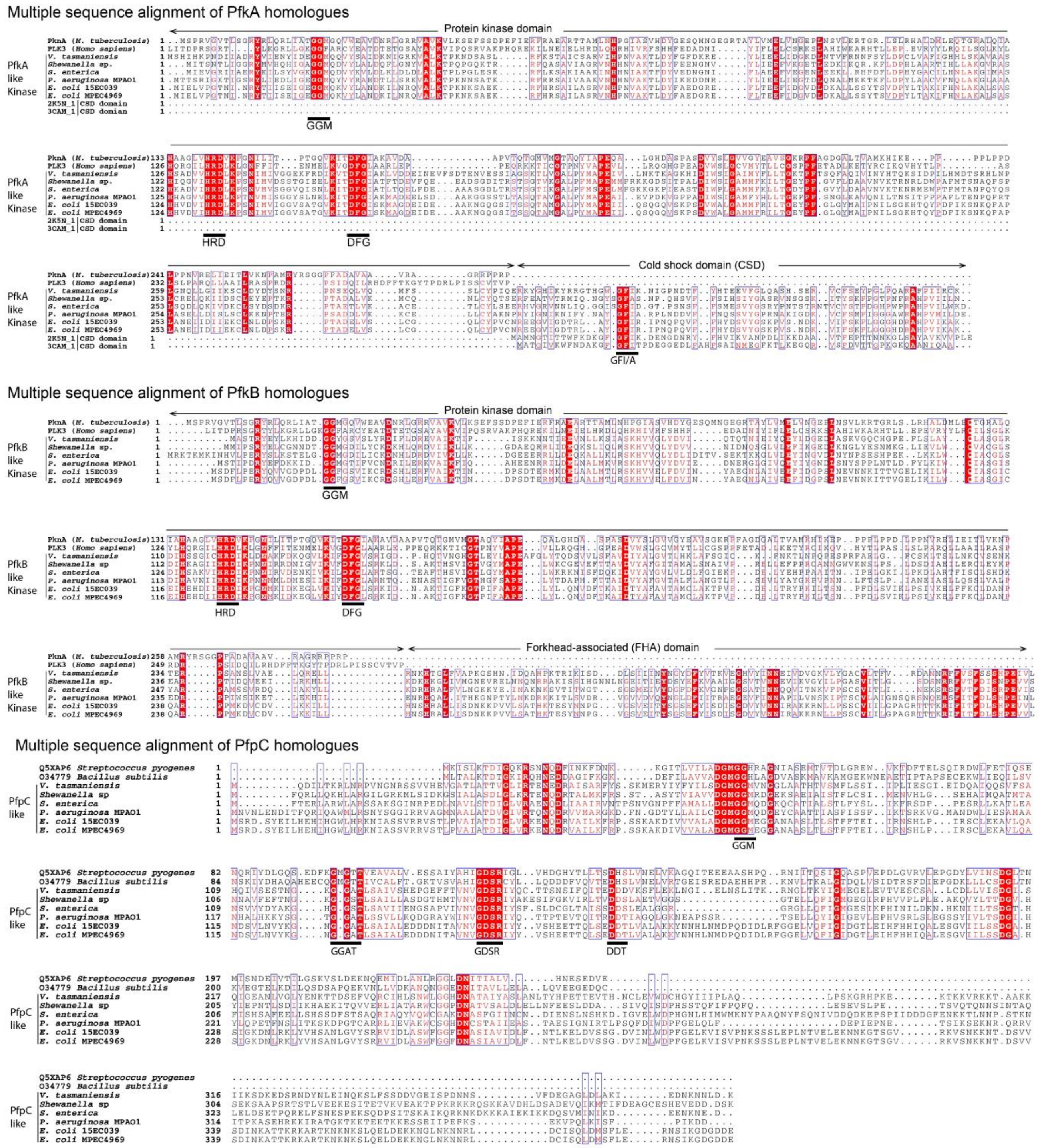

Supplementary Fig. 2 Sequence comparisons and predicted structures of the components of MPAO1 KKP module.

Sequence alignment of PfkA and PfkB homologues. Homologues of PfkA and PfkB used in this study were aligned with Ser/Thr kinase PknA of *Mycobacterium tuberculosis* (NCBI accession number NP_214529) and Ser/Thr kinase PLK3 of *Homo sapiens* (NCBI accession number NP_004064). Accession numbers of PfkA and PfkB homologues from *Vibrio tasmaniensis* 10N.222.48. A2, *Shewanella* sp. W3-18-1, *Salmonella enterica* enterica sv. Typhi CT18 and *E. coli* strains are listed in Supplementary Data 2. The conserved domains mutated in Fig. 4a and Supplementary Fig. 6a are underlined.

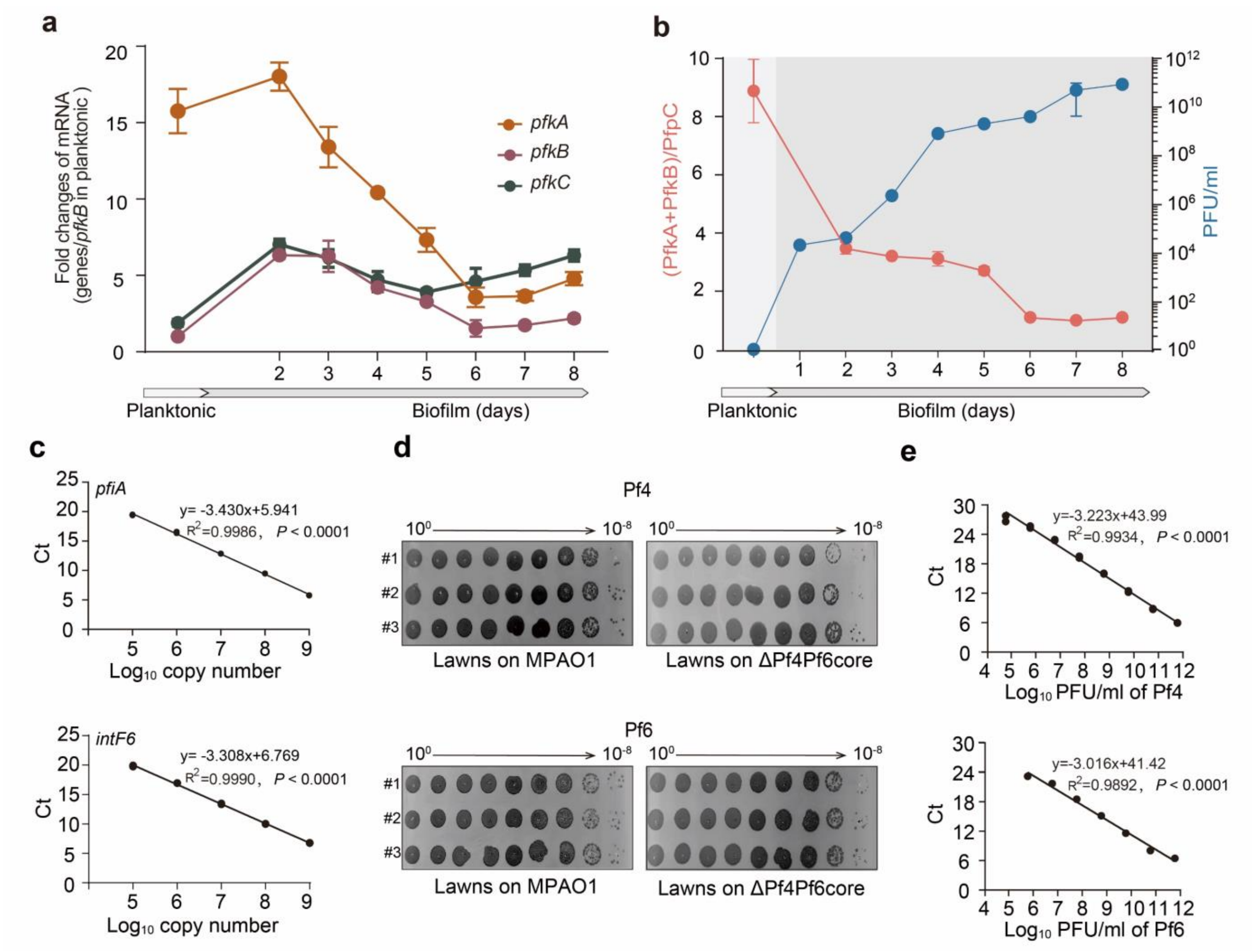

Supplementary Fig. 3 Expression dynamics of KKP_{MP} genes via qRT-PCR and quantification of Pf4 and Pf6 phages via qPCR.

a, All the genes determined in MPAO1 planktonic and biofilm cells (day 2-8) were normalized to 16S rRNA and the fold changes of mRNA were calculated as compared to *pfkB* in planktonic cells.

b, Kinetic analysis of Pf phage release and the mRNA ratio of kinases versus phosphatase (*pfkA*+*pfkB)*/*pfpC*.

c, Amplification efficiencies of Pf4-specific *pfiA* and Pf6-specific *intF6* primers with serial dilution of pMD19-*pfiA* and pMD19-*intF6* plasmids via qPCR.

d, Phage titers of Pf4 and Pf6 used for qPCR.

e, Pf4 and Pf6 phage dilutions shown in **d** were used for qPCR, and the correlation between PFU/ml and Ct values was analyzed.

Three independent replicates were used for all experiments, and data were shown as the mean ± SD in **a-c** and **e**.

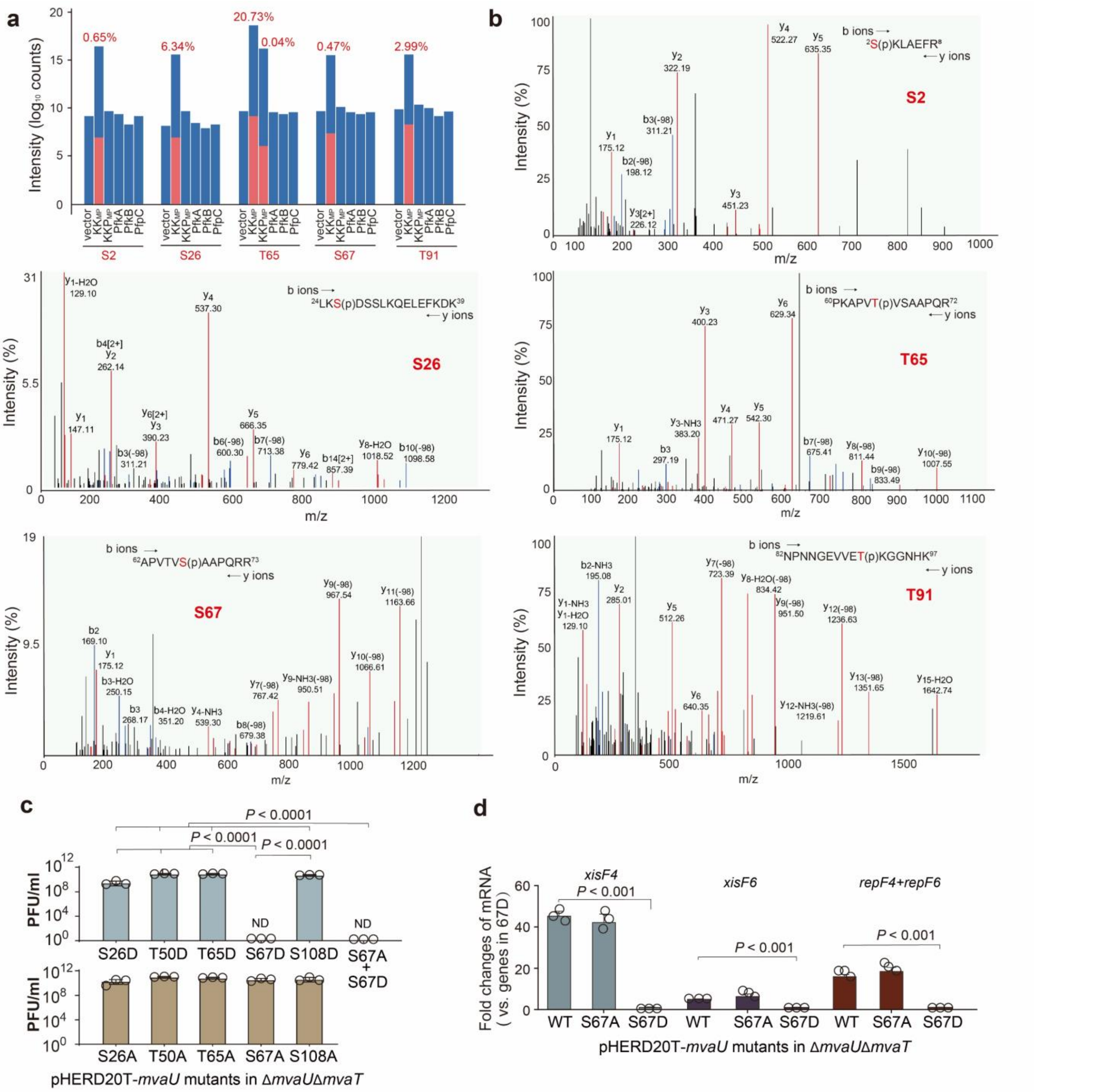

Supplementary Fig. 4 MvaU is phosphorylated at multiple sites and phosphorylation of S67 in MvaU inhibits Pf phage production.

a, Chromosomal His-tagged MvaU was purified and phosphorylated sites were identified by mass spectrum when indicated KKP_{MP} components were overexpressed. Purified MvaU-His protein was subjected to LC–MS/MS analysis after trypsin digestion. The number above the columns indicates the phosphorylation percentages of each site in different hosts.

b, MS/MS spectrum of five phosphorylated tryptic MvaU-His peptides include S2, S26, T65, S67 and T91 when PfkA/B were overexpressed. The b and y fragment ions of the peptide sequence are labeled in the spectrum. S(p) and T(p) indicate the phosphorylated Ser and Thr residues, respectively.

c, Planktonic Pf4 phage titers from indicated MvaU variants expressed in MPAO1 Δ*mvaU*Δ*mvaT*.

d, Effects of MvaU WT or $MvaU^{S67D}$ on the expression of Pf4 and Pf6 excision and replication-related genes in planktonic MPAO1 Δ*mvaU*Δ*mvaT* cells.

Data in **c** and **d** are shown as the mean ± SD. Ordinary one-way ANOVA with Tukey's multiple comparisons test was used (n=3), $P < 0.05$ was considered statistically significant.

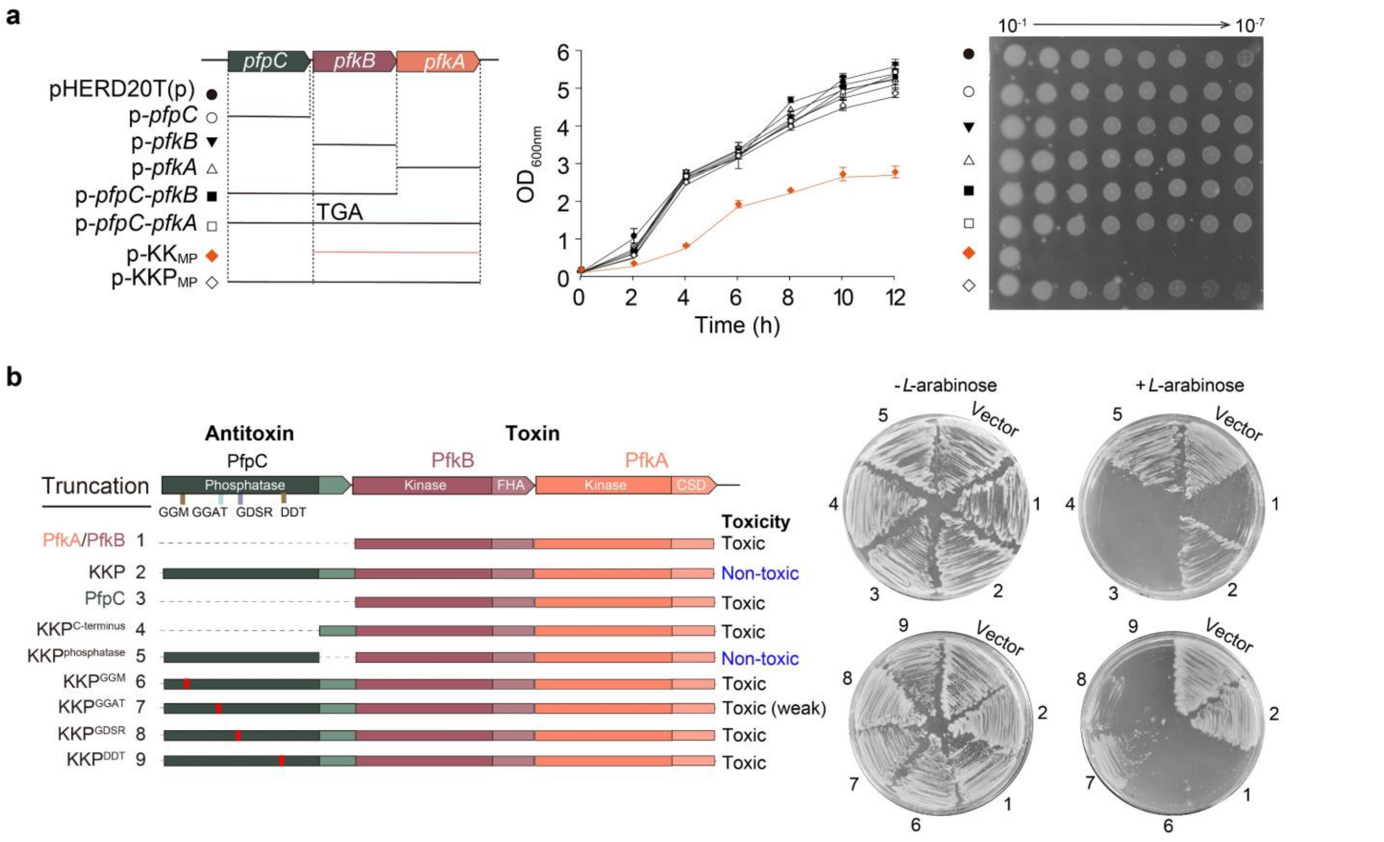

Supplementary Fig. 5 KKP$_{MP}$ is a toxin/antitoxin system.

a, Schematic of constructs used for introduction of KKP$_{MP}$ components via pHERD20T into *P. aeruginosa* PAO1 (left). TGA indicates where the start codon of PfkB was replaced by a stop codon to prevent PfkB translation. Toxicity of introduced KKP$_{MP}$ components was assessed with growth in liquid media (middle) with *L*-arabinose for 12 h and CFU were assessed at 6 h (right).

b, Toxicity of KKP$_{MP}$ TA system with phosphatase domain and/or C-terminal domain of unknown function mutated. Deletion of the PfpC C-terminal domain did not affect the ability to neutralize the toxicity of KK$_{MP}$, while mutating the phosphatase domain reduced the neutralization ability.

Three independent replicates were used and only representative images were shown here.

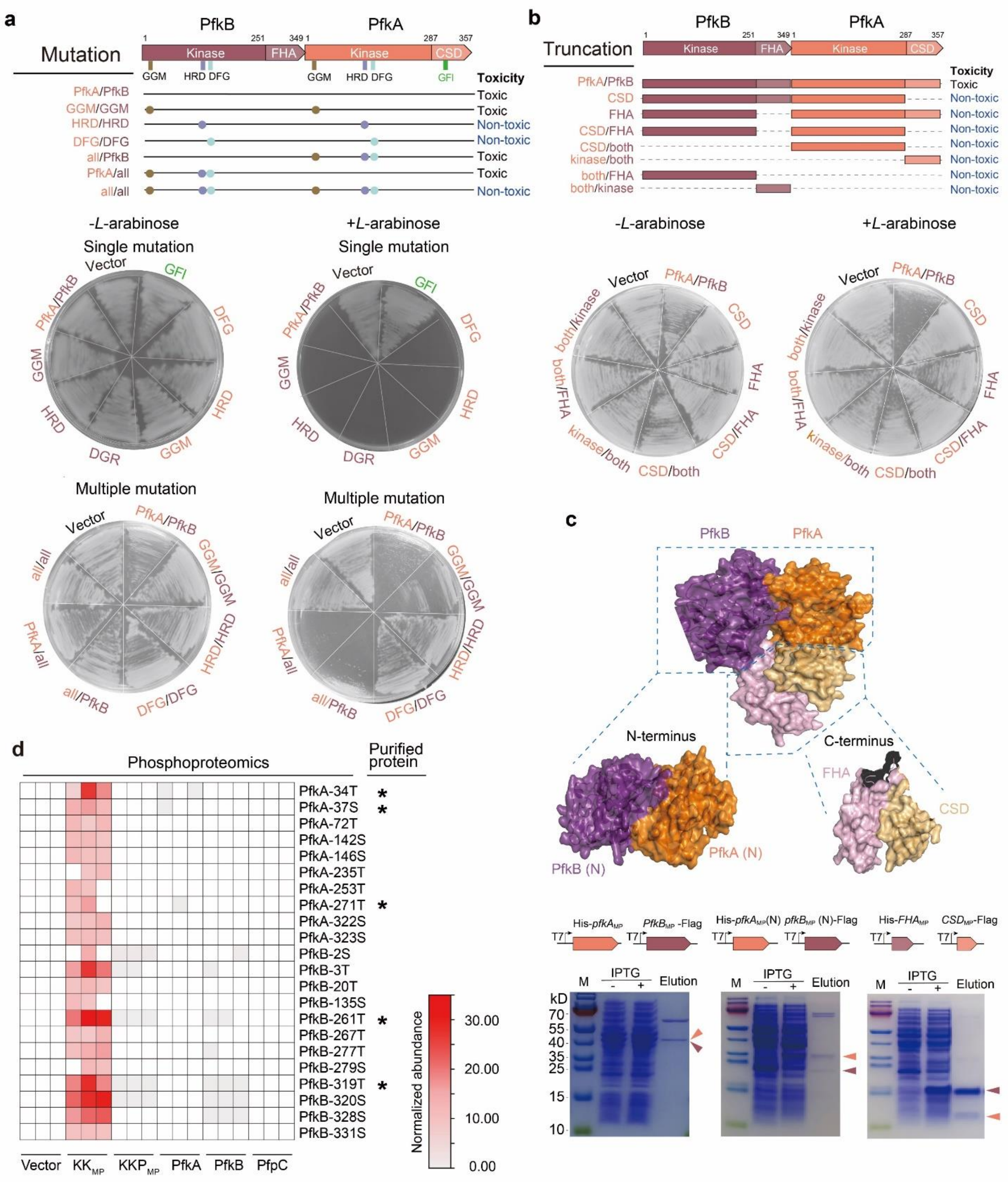

Supplementary Fig. 6 PfkA interacts with PfkB and PfpC neutralizes PfkA/PfkB toxicity.

a, Schematic of KKP_{MP} components with conserved kinase motifs in PfkA and/or PfkB mutated (top). Toxicity of cells harboring single GFI mutant in PfkA or two HRDs or two DFGs mutations in both PfkA and PfkB or all three conserved motifs in both PfkA and PfkB abolished toxicity in the presence of inducer *L*-arabinose (below).

b, Schematic of KKP_{MP} components containing truncated PfkA and PfkB (top). The C-terminus of PfkA or PfkB is important for toxicity.

c, Top: Alphafold2 prediction of the heterodimer structure of PfkA-PfkB complex. The kinases likely interact between their respective kinase and CSD and FHA domains. Bottom: pulldown assays *in E. coli* heterologously expressing the indicated constructs. The identities of the bands in the elution were confirmed by mass spectrometry. Arrows indicate bands at the expected size of the respectively colored constructs.

d, Heat map showing a subset of the most highly phosphorylated sites in PfkA and PfkB with KKP_{MP} components expressed in PAO1. The phosphorylated sites of purified PfkA-His and PfkB-His with KK_{MP} overproduction were confirmed by MS, and were shown in asterisks.

Three independent biological replicates were tested for **a**, **b**, **d** and representative images were shown for **a**-**c**.

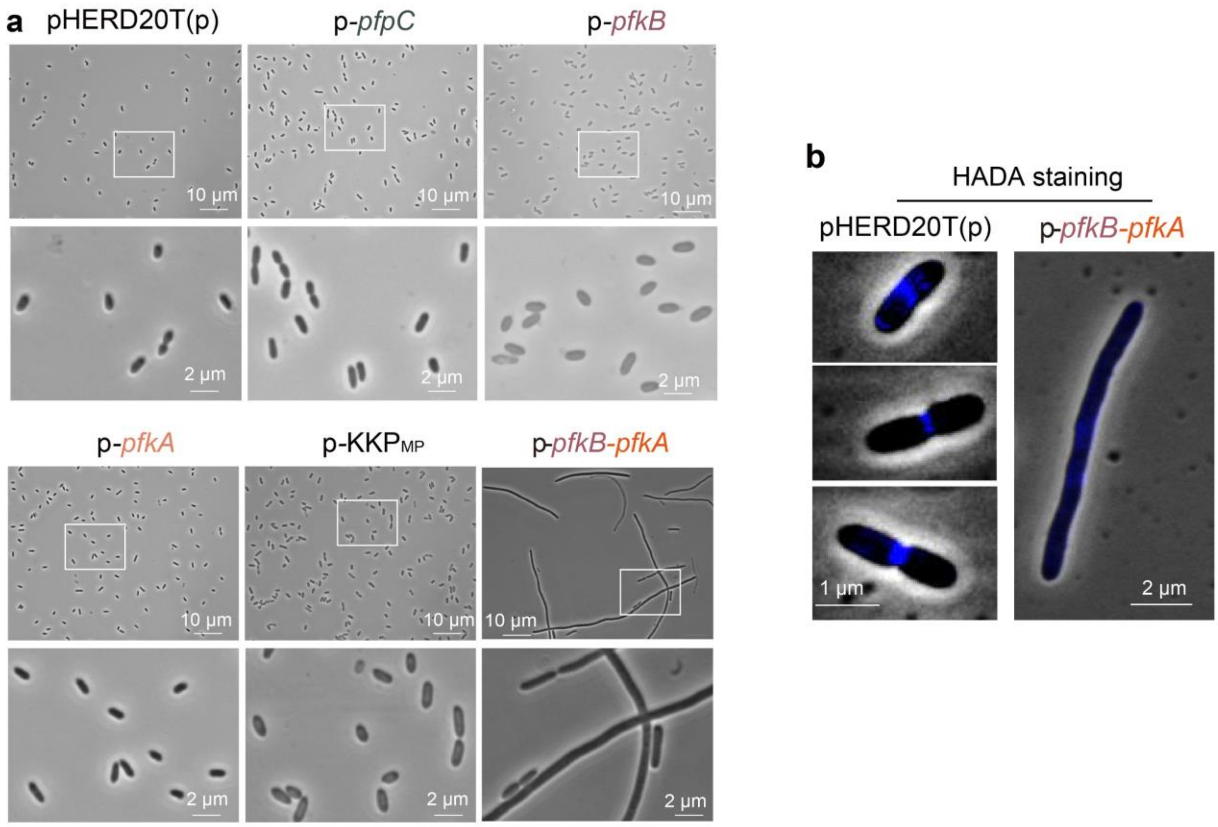

Supplementary Fig. 7 Cell morphology of the overexpressed KKP_{MP} components.

a, Cell morphology of the indicated PAO1/pHERD20T expressing of KKP_{MP} components for 6 h. The enlarged image on the bottom was from the boxed region on the top panel.

b, Microscopy of HADA-stained cells with PAO1/pHERD20T expressing *pfkB-pfkA* for 6 h.

Three independent cultures were used for all the experiments and only representative images were shown.

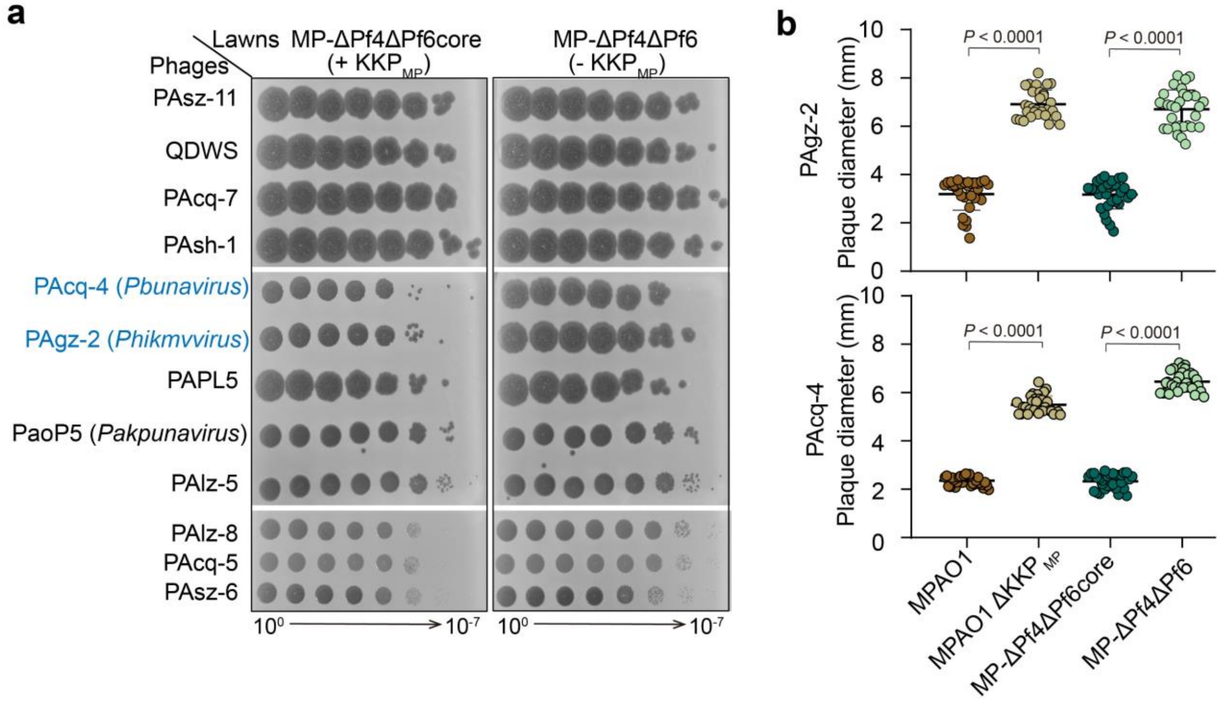

Supplementary Fig. 8 KKP_{MP} provides phage defense by reducing plaque sizes.

a, Sensitivity of MP-ΔPf4ΔPf6core and MP-ΔPf4ΔPf6 to different *P. aeruginosa* phages. PAcq-4 and PAgz-2 reduced plaque sizes.

b, Diameters of plaques in Fig. 5e. All data dots are shown, and two-sided Student's *t*-Test was used for comparisons between two indicated groups in the presence of PAgz-2 or PAcq-4 lytic phages (*n*=30). $P < 0.05$ was considered statistically significant.

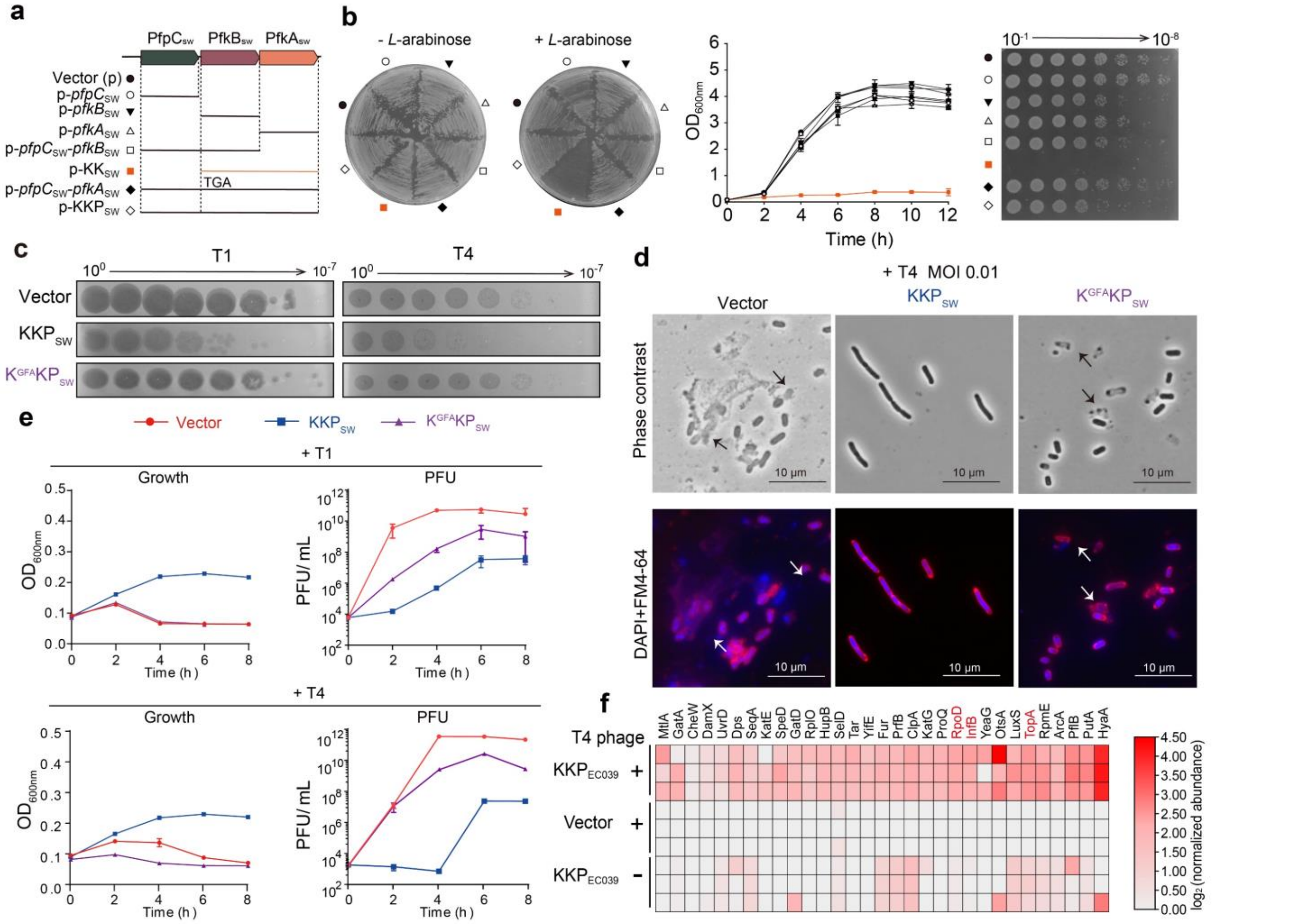

Supplementary Fig. 9 Further characterization of KKP$_{SW}$ and KKP$_{EC039}$.

a, Schematic of KKP$_{SW}$ component constructs introduced via pHERD20T into *E. coli*.

b, Toxicity of KKP$_{SW}$ components in *E. coli* was assessed with LB agar plates and liquid growth (left and middle) media-based assays with *L*-arabinose for 12 h and CFU were assessed at 6 h (right).

c, Sensitivity of *E. coli* cells harboring KKP$_{SW}$ and K^{GFA}KP$_{SW}$ mutant to T1 and T4 phages.

d, Microscopy of *E. coli* cells expressing KKP$_{SW}$, and K^{GFA}KP$_{SW}$ after infection with T4 at MOI of 0.01; red, FM4-64; blue, DAPI; arrows indicate lysed cells.

e, Growth curves for *E. coli* harboring an empty vector or plasmid-encoded KKP$_{SW}$ or K^{GFA}KP$_{SW}$ after infection with phages T1 or T4 at MOI of 0.1. PFU was also determined at indicated time points after infection with T1 and T4.

f, Heatmap shows a subset of the most highly phosphorylated proteins in the presence of KKP$_{EC039}$ during T4 phage attack. Abundances of phosphorylated proteins from *E. coli* hosts expressing KKP$_{EC039}$ via plasmid are shown. *E. coli* proteins known to support phage propagation are highlighted in red.

Three independent biological replicates are used in **b**-**f** and data in **b** are shown as the mean ± SD, representative images were shown in **b**-**d**.

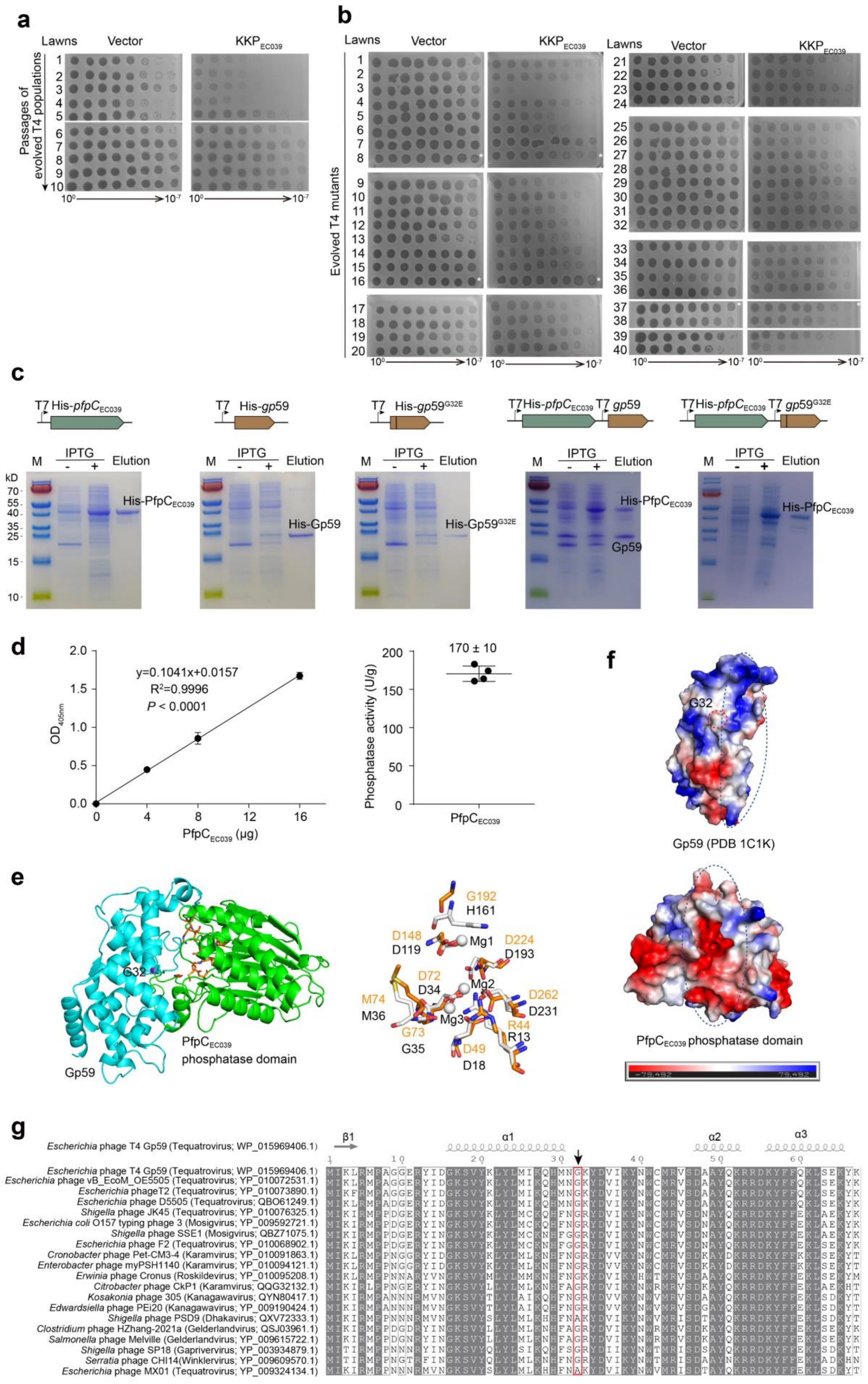

Supplementary Fig. 10 Evolved T4 phages escape KKP$_{EC039}$ defense.

a, Serial dilutions of T4 populations at different passages (1-10) evolved in the presence of KKP$_{EC039}$. Antiphage activity against evolved phages was determined using *E. coli* expressing KKP$_{EC039}$.

b, Infection of *E. coli* expressing KKP$_{EC039}$ by individual evolved T4 phages at passage 10.

c, Co-elution of N-terminal His-tagged PfpC$_{EC039}$ or Gp59 with the indicated co-expressed protein in *E. coli*.

d, pNPP phosphatase assay standard curve with serial dilution of $PfpC_{EC039}$ at 10 minutes (left). Phosphatase activity was calculated with 10 µg $PfpC_{EC039}$ (right).
e, Alternate view of predicted Gp59-$PfpC_{EC039}$ phosphatase domain complex. $Gp59^{G32}$ is displayed in purple and $PfpC_{EC039}$ active site residues are displayed in orange (left). The active site of PfpC was identified by comparison to the characterized PP2C family phosphatase tPphA of *Thermosynechococcus elongatus* (PDB 2J86, right overlay).
f, Surface electrostatic potentials of Gp59 (PDB 1C1K, left) and $PfpC_{EC039}$ phosphatase domain (right). G32 residue is circled by the dashed red ovals, and the potential interaction surface of Gp59 and $PfpC_{EC039}$ is circled by the dashed blue ovals.
g, Multiple sequence alignment of Gp59 homologs from different phages. The G32 is indicated with an arrow. Phage genera and accession numbers of Gp59 homologs are shown on the left.
Two independent experiments were conducted for **c,** and at least three independent replicates were used in **d** (*n*=3 for left panel and n=4 for right panel) and data were shown as the mean ± SD.

Supplementary Tables

Supplementary Table 1. **List of the Pf4 and Pf6 prophage genes in MPAO1 and their genomic locations**. The core prophage genes are highlighted in bold. Corresponding locus tags in PAO1 and similarity metrics are listed for Pf4 genes.

Pf4 in MPAO1 (12416 bp)						Pf6 in MPAO1 (12150 bp)					Nucleotide		Protein	
Locus tag (MPAO1)	Locus tag (PAO1)	Gene name	Start	Stop	Strand	Locus tag (MPAO1)	Gene name	Start	Stop	Strand	Coverage	Identity	Coverage	Identity
		attR	4717479	4717505	-									
MP_4487	PA0729	*pfiT*	4717628	4717975	-									
MP_4488	PA0728.1	*pfiA*	4717985	4718236	-		*attR*	5241438	5241519					
MP_4489	PA0728	*intF4*	4718450	4719433	-	MP_4975	*intF6*	5241680	5242681	-	0	0	98%	40.66%
**MP_4490**	**PA0727**	***repF4***	**4719433**	**4720725**	-	**MP_4976**	***repF6***	**5242678**	**5243889**	-	**96%**	**92.30%**	**93%**	**95.29%**
**MP_4491**	**PA0726.1**		**4720857**	**4720958**	-	**MP_4977**		**5244102**	**5244203**	-	**100%**	**97.06%**	**100%**	**93.94%**
**MP_4492**	**PA0726**		**4720955**	**4722229**	-	**MP_4978**		**5244200**	**5245474**	-	**100%**	**98.27%**	**100%**	**97.88%**
**MP_4493**	**PA0725**	***gVI***	**4722233**	**4722589**	-	**MP_4979**	***gVI***	**5245478**	**5245834**	-	**100%**	**99.16%**	**100%**	**100%**
**MP_4494**	**PA0724**	***gIII***	**4722594**	**4723856**	-	**MP_4980**	***gIII***	**5245839**	**5247098**	-	**100%**	**98.10%**	**100%**	**98.81%**
**MP_4495**	**PA0723**	***gVIII***	**4723992**	**4724240**	-	**MP_4981**	***gVIII***	**5247234**	**5247482**	-	**100%**	**99.20%**	**100%**	**100%**
**MP_4496**	**PA0722**	***gIX***	**4724253**	**4724504**	-	**MP_4982**	***gIX***	**5247495**	**5247746**	-	**100%**	**100%**	**100%**	**100%**
**MP_4497**	**PA0721**	***gVII***	**4724517**	**4724609**	-	**MP_4983**	***gVII***	**5247759**	**5247851**	-	**100%**	**97.85%**	**100%**	**96.67%**
**MP_4498**	**PA0720**		**4724626**	**4725060**	-	**MP_4984**		**5247868**	**5248302**	-	**100%**	**96.09%**	**100%**	**97.92%**
**MP_4499**	**PA0719**		**4725195**	**4725572**	-	**MP_4985**		**5248469**	**5248813**	-	**99%**	**97.38%**	**79%**	**98.99%**
**MP_4500**	**PA0718**		**4725576**	**4725866**	-	**MP_4986**		**5248817**	**5249107**	-	**100%**	**96.91%**	**100%**	**96.88%**
**MP_4501**	**PA0717**		**4725870**	**4726082**	-	**MP_4987**		**5249111**	**5249239**	-	**60%**	**97.67%**	**60%**	**97.62%**
MP_4502	PA0716.2	*xisF4*	4726084	4726299	-	MP_4988	*xisF6*	5249319	5249549	-	0	0	49%	34.29%
MP_4503	PA0716.1	*pf4r*	4726418	4726684	+	MP_4989	*pf6r*	5249717	5249986	+	0	0	100%	50.56%
MP_4504	PA0716		4726973	4728298	-	MP_4990	*pfpC*	5250121	5251218	+				
MP_4505	PA0715		4728301	4729257	-	MP_4991	*pfkB*	5251221	5252270	+				
MP_4506	PA0714.1	*phrD*	4729635	4729707	-	MP_4992	*pfkA*	5252267	5253340	+				
		attL	4729868	4729894	-		*attL*	5253510	5253589					

Supplementary Table 2. Presence of Pf4 and Pf6 loci in the selected *P. aeruginosa* strains as analyzed by BLASTN. The four genomes used in Fig. 1d are shown in blue fonts and two ATCC strains were shown in bold fonts. In addition, the strains with determined sources summarized by C. E., Chandler et al.[1] were also analyzed, and the assembly of these strains were downloaded from NCBI under BioProject accession number PRJNA490649. Y indicates yes and N indicates no.

GenBank/Assembly ID	Strain name	Pf4	Pf6	Source	Date acquired	PubMed ID	Date published
**MPAO1 sublines**							
**CP079712**	**MPAO1**	**Y**	**Y**	**McDermott, University of Washington**	**07/2020**	**this study**	
**CP027857**	**MPAO1 (C. Manoil)**	**Y**	**Y**	**C. Manoil, University of Washington**	**2017**	**33127897**	2020 Oct 30
GCA_003957075	MPAO-1 (C. Manoil)	Y	Y	C. Manoil, University of Washington	01/2003	30530517	2019 Feb 11
GCA_003957195	MPAO-1 (C. Manoil)	Y	Y	C. Manoil, University of Washington	08/2008	30530517	2019 Feb 11
GCA_003957175	PAO-1 (B. Iglewski)	Y	Y	E.P. Greenberg, University of Washington	10/2000	30530517	2019 Feb 11
CP050052	PAO1 (B. Iglewski)	Y	Y	E. P. Greenberg, University of Washington	04/2019	unpublished	
GCF_003957105	H103 PAO-1 AK957	Y	Y	R. Hancock, University of British Columbia	02/2000	30530517	2019 Feb 11
GCA_003957205	PAO-1 (B. Holloway)	Y	Y	D. Ohman, Virginia Commonwealth University	05/2001	30530517	2019 Feb 11
GCA_003957095	PAO-1 V	Y	Y	J. Goldberg, University of Virginia	07/2003	30530517	2019 Feb 11
GCA_003957085	PAO-1	Y	Y	H. Nikaido, University of California, Berkeley	08/2003	30530517	2019 Feb 11
GCA_003957115	PAO-1	Y	Y	A. Prince, Columbia University	11/2003	30530517	2019 Feb 11
CP050054/CP050053	PAO1	Y	Y	Y. Liu, South China Agricultural University	04/2018	unpublished	
CP053028	PAO1	Y	Y	S.P. Diggle, Georgia Institute of Technology	01/2014	32341475	2020 Mar 23
not available	PAO1	Y	Y	Scott A. Rice, Nanyang Technological University	not available	34452479	2021 Aug 15
LN871187	PAO1_Orsay1	Y	Y	Pourcel, Institut de Genetique et Microbiologie	not available	unpublished	
CP032126	PAO1161	Y	Y	A. Kawalek, Institute for Biochemistry and Biophysics PAS	11/2017	31906858	2020 Jan 6
**NZ_CP017149**	**PAO-1C (ATCC 15692)**	**Y**	**Y**	**ATCC (deposited by BW Holloway)**	**not available**	unpublished	
**CP041008**	**PAO1 (ATCC BAA-47)**	**Y**	**Y**	**ATCC (deposited by HW Ackermann)**	**not available**	unpublished	
**PAO1 sublines**							
**CP085082**	**PAO1**	**Y**	**N**	**J.J. Mekalanos, Harvard Medical School**	**10/2020**	**this study**	
**AE004091**	**PAO1**	**Y**	**N**	**P. Phibbs, University of Georgia**	**not available**	**10984043**	**2000 Aug 31**
GCA_003957185	PAO-1	Y	N	J. Burns, University of Washington	02/2000	30530517	2019 Feb 11
CP006832/CP006831	PAO1	Y	N	H.W.D., Yu, Marshall University	not available	24336371	2013 Dec 12
CP006705	PAO1 (PA0581)	Y	N	H.W.D., Yu, Marshall University	not available	24115549	2013 Oct 10
CP053110 to CP053119	PAO1	Y	N	S. Fong, University of Adelaide	12/2017	unpublished	
CP047061 to CP047068	PAO1	Y	N	Balint Csorgo, UCSF	not available	unpublished	
CP032540/CP032541	PAO1	Y	N	Progenesis Technologies, LLC	09/2014	unpublished	
CP034908	PA0750	Y	N	H.P. Schweizer, Colorado State University	2005	17005832	2006 Oct

Supplementary Table 3. **RNA-seq reads of each Pf4 and Pf6 genes in biofilm and planktonic MPAO1 cells.** Three replicates were used and shown. NA indicates not available. FC, CPM and FDR indicate fold change, counts per million and false discovery rate, respectively.

Locus tag (MPAO1)	Gene product	Planktonic cells			Biofilm cells			LogFC	LogCPM	FDR
		# 1	# 2	# 3	# 1	# 2	# 3			
**Pf4 prophage**										
**MP_4487**	PfiT	59.0	69.9	45.6	583.1	272.6	468.9	3.0	6.4	5.29E-36
**MP_4488**	PfiA	60.2	47.0	63.6	539.2	302.2	369.8	2.9	5.9	1.55E-24
**MP_4489**	IntF4	1.7	4.0	2.3	459.7	103.6	155.5	6.5	6.8	2.72E-44
**MP_4490**	RepF4	2.9	3.8	3.6	892.8	196.0	316.5	7.1	8.2	6.83E-54
**MP_4491**	Hypothetical	0.0	0.0	0.0	41.5	1.8	9.3	6.3	0.1	0.00088
**MP_4492**	Hypothetical	1.0	0.6	1.0	576.3	122.4	177.4	8.3	7.5	8.38E-53
**MP_4493**	pVI	0.0	0.9	0.0	23.7	3.0	0.5	4.7	0.8	0.001108
**MP_4494**	pIII	0.3	1.2	1.3	1196.3	249.6	426.0	9.4	8.5	4.14E-62
**MP_4495**	pVIII	1.3	4.3	6.4	11773.3	1518.9	1889.6	10.2	9.2	2.20E-35
**MP_4496**	pIX	0.0	0.0	0.0	0.0	0.0	0.0	NA	NA	NA
**MP_4497**	pVII	0.0	0.0	0.0	123.4	15.5	34.5	7.9	1.4	1.12E-08
**MP_4498**	Hypothetical	8.3	23.8	9.2	20618.8	4612.1	8445.6	9.7	11.2	5.79E-77
**MP_4499**	Hypothetical	8.6	9.7	0.9	9635.9	2351.2	4761.3	9.8	10.0	1.07E-71
**MP_4500**	Hypothetical	3.4	4.7	8.8	5423.0	2356.9	3570.8	9.5	9.1	8.37-126
**MP_4501**	Hypothetical	0.8	0.7	3.0	1681.4	588.2	744.9	9.4	6.7	7.87E-83
**MP_4502**	XisF4	2.3	1.4	2.2	5079.5	950.1	1656.8	10.3	8.0	1.58E-62
**MP_4503**	Pf4r	24.4	23.9	18.6	48.3	58.0	110.9	1.9	3.7	1.36E-06
**MP_4504**	Hypothetical	48.2	38.2	32.7	39.9	68.0	94.3	0.99	6.2	0.01418
**MP_4505**	Hypothetical	48.4	22.9	23.4	33.9	60.2	91.3	1.2	5.6	0.01255
**MP_4506**	PhrD	464.6	878.3	875.3	391.6	254.0	434.2	-0.92	5.3	0.00379
**Pf6 prophage**										
**MP_4975**	IntF6	4.9	2.3	3.8	61.9	93.2	142.0	4.9	5.8	9.62E-27
**MP_4976**	RepF6	3.2	2.5	2.1	164.9	237.5	393.0	6.9	7.5	4.69E-49
**MP_4977**	Hypothetical	0.0	0.0	0.0	63.2	88.2	160.9	9.1	2.5	1.01E-18
**MP_4978**	Hypothetical	0.1	0.6	0.5	70.1	135.1	200.5	8.6	6.6	1.07E-44
**MP_4979**	pVI	0.0	0.0	0.0	10.7	9.1	9.5	7.5	1.0	2.15E-09
**MP_4980**	pIII	1.0	0.5	1.0	189.8	335.9	525.1	8.9	7.9	1.92E-52

**MP_4981**	pVIII	0.0	1.2	2.6	3837.9	2455.9	2303.2	11.2	8.5	8.44E-126
**MP_4982**	pIX	0.0	0.0	0.0	0.0	0.0	0.0	NA	NA	NA
**MP_4983**	pVII	0.0	0.0	0.0	10.8	7.7	24.3	6.0	-0.1	0.000402
**MP_4984**	Hypothetical	4.1	6.3	4.4	4002.7	4457.3	6895.2	10.2	10.2	1.91E-110
**MP_4985**	Hypothetical	3.8	4.4	0.9	4505.9	4766.5	8160.2	11.1	10.1	4.38E-108
**MP_4986**	Hypothetical	0.6	1.0	2.2	2020.1	2039.6	3080.0	11.0	8.5	8.22E-108
**MP_4987**	Hypothetical	2.5	0.0	2.5	1373.3	1618.7	1675.9	10.0	6.8	4.59E-81
**MP_4988**	XisF6	3.5	4.6	1.4	4576.2	6590.4	10843.9	11.4	9.9	1.21E-85
**MP_4989**	Pf6r	186.0	146.3	99.1	1427.1	462.0	666.6	2.6	7.0	8.03E-17
**MP_4990**	PfpC	93.1	67.4	46.4	468.6	209.2	306.5	2.3	7.8	1.02E-16
**MP_4991**	PfkB	162.3	109.5	76.3	2198.4	953.6	1415.6	3.8	9.7	3.64E-34
**MP_4992**	PfkA	731.0	674.1	431.5	933.8	319.8	466.5	-0.02	9.3	0.97

Supplementary Table 4. List of putative PfkA kinase substrates identified by phosphoproteomics. The fold changes indicate the phosphorylation levels of proteins controlled by PfkA via pHERD20T-*pfkA* overproduction versus those in cells harboring pHERD20T empty vector. Two replicates were used, and those proteins with fold changes > 2.5 and coefficient of variation (CV) < 0.1) were selected.

**Locus tag (MPAO1)**	**Locus tag (PAO1)**	**Protein**	**Position**	**Fold change**	**CV value**	**Description**
MP_2627	PA2480		391T	4.554	0.006	Signal transduction histidine kinase
MP_4036	PA1155	NrdB	408T	3.968	0.008	Ribonucleotide-diphosphate reductase subunit beta
MP_618	PA0577	DnaG	527T	3.816	0.088	DNA primase
MP_1549	PA3481	ApbC	353S	3.777	0.022	ATPases involved in chromosome partitioning
MP_3583	PA1585	SucA	62T	3.616	0.072	Dehydrogenase (E1) component related enzymes
MP_82	PA0074	PpkA	270T	3.261	0.087	Ser/Thr kinase
MP_723	PA4274	RplK	71T	3.101	0.046	Ribosomal protein L11
MP_5588	PA5239	Rho	84S	3.010	0.060	Transcription termination factor
MP_2467	PA2629	PurB	296S	2.975	0.088	Adenylosuccinate lyase
**MP_2427**	**PA2667**	**MvaU**	**65T/67S**	**2.892**	**0.019**	**H-NS family protein**
MP_5084	PA4759	DapB	207T	2.640	0.069	Dihydrodipicolinate reductase
MP_4426	PA0787		79S	2.624	0.089	Predicted ATPase
MP_5631	PA5279		49S	2.609	0.085	Uncharacterized protein conserved in bacteria
MP_5697	PA5343		246S	2.604	0.026	Nucleoside-diphosphate-sugar epimerases
MP_5797	PA5435	PycB	462T	2.517	0.022	Pyruvate/oxaloacetate carboxyltransferase
MP_1500	PA3529		198S	0.229	0.049	Peroxidase
MP_1153	PA3861	RhlB	38S	0.129	0.044	ATP-dependent RNA helicase RhlB
MP_180	PA0170		68T	0.017	0.083	DUF1987 domain-containing protein

Supplementary Table 5. Mass spectrometry results of purified PfkA$_{MP}$-His during co-purification of PfkA$_{MP}$-His and PfkB$_{MP}$ (refer to **Supplementary Fig.6c left panel**, Elution). Peptide fragments identified by mass spectrometry analysis are listed and correspond to the highlights in the PfkA$_{MP}$-His protein sequence below. For the PfkA$_{MP}$-His protein, the -10lgP is 435.54.

Peptide	-10lgP	Mass	Length	ppm	m/z	z	RT	Area
RYLIEDLIGEGGMQYVYRA	107.42	2017.9818	17	1.4	1009.9996	2	96.73	6.89E8
KGLAAAHHAGVVHRD	105.77	1294.7007	13	0.9	648.3582	2	12.40	9.98E8
KAVQNILTNSITPPPAFLTENPQFRT	103.93	2667.4021	24	1.0	1334.7097	2	90.59	1.38E9
RPLNDDVFFNQSSVYGPRN	102.17	1953.9220	17	1.7	977.9699	2	74.19	9.92E8
KTLDYIKEDDNRY	94.89	1380.6521	11	0.4	691.3336	2	30.27	1.49E8
RDLKPTNVMISGGYSLNELKI	88.62	2078.0718	19	0.5	1040.0437	2	69.23	3.88E8
RTLASELLDISLSCLKK	88.42	1661.8910	15	-0.2	831.9526	2	105.77	1.09E8
RAHDTLLSRK	83.13	991.4488	8	-0.3	496.7315	2	22.01	3.07E8
KMADEELSDAAKG	81.33	1178.5125	11	0.0	590.2635	2	29.81	2.41E8
KGGSSTLTASQTAVGALPYMAPEAIEVPDKV	76.93	2860.4163	29	2.9	954.4821	3	81.75	6.55E7
RAHPVMILDEHHHHHH	75.77	1845.8594	15	1.6	462.4728	4	18.91	5.61E9
YGFIARPLNDDVFFNQSSVYGPRN	74.35	2498.2341	22	0.9	833.7527	3	76.29	1.37E7
KKCELLCYAKN	73.98	1183.5729	9	0.9	592.7943	2	34.13	9.2E6
RIGKTIGSRY	69.64	1067.5498	10	0.3	534.7823	2	33.79	1E7
KNIFYNAYGFIARP	67.36	1447.7247	12	-0.4	724.8693	2	82.46	2.03E8
CFSMFIGGGAWRA	65.12	1227.5858	11	0.8	614.8007	2	78.19	4.5E6
KIGDKVCFSMFIGGGAWRA	63.40	1915.9072	17	1.1	639.6437	3	74.15	8.25E6
KFLDPYLAACVFNQLAKG	61.50	1868.9495	16	0.5	623.9907	3	101.47	8.07E7
RYLIEELIEGTDLQNALMKK	46.40	2108.0710	18	-0.2	1055.0426	2	101.82	3.71E6
ITDFGIAKM	29.15	750.3912	7	1.1	751.3993	1	40.64	6.66E7
MFYHLLTGEFPFSNGLKA	26.55	1868.9460	16	2.0	623.9905	3	81.11	1.22E6
KNPTERPSADDL	23.69	1100.4734	10	-0.6	551.2437	2	11.00	1.36E6

363 aa of PfkA$_{MP}$-His
MTTSRIGKTIGSRYLIEDLIGEGGMQYVYRAHDTLLSRKVALKTPKNNSATKRFRRSAIVAAKVNHHNVAKTLDYIKED
DNRYLIEELIEGTDLQNALMKKTKFLDPYLAACVFNQLAKGLAAAHHAGVVHRDLKPTNVMISGGYSLNELKITDFGIA
KMADEELSDAAKGGSSTLTASQTAVGALPYMAPEAIEVPDKVGPPADIWSIGAMFYHLLTGEFPFSNGLKAVQNILTNS
ITPPPAFLTENPQFRTLASELLDISLSCLKKNPTERPSADDLVKKCELLCYAKNPRYIGNIKNIFYNAYGFIARPLNDDV
FFNQSSVYGPRNAKIGDKVCFSMFIGGGAWRAHPVMILDEHHHHHH

Supplementary Table 6. Mass spectrometry results of the purified protein Flag-PfkB$_{MP}$ during co-purification of His-PfkA$_{MP}$ and Flag-PfkB$_{MP}$ (refer to **Supplementary Fig.6 left panel**, Elution). Peptide fragments identified by mass spectrometry analysis are listed and correspond to the highlights in the PfkB$_{MP}$-Flag protein sequence below. For the Flag-PfkB$_{MP}$ protein, the -10lgP is 510.60.

Peptide	-10lgP	Mass	Length	ppm	m/z	z	RT	Area
KIWQIASGISDIHAVNIIHRD	130.34	2142.1697	19	1.2	1072.0934	2	72.06	1.42E9
RSKHVVQVYDVIEDNERG	115.17	1928.9591	16	1.6	965.4884	2	43.49	2.57E8
KIPTSCVLAIGNSQRS	109.99	1594.7538	14	0.7	798.3847	2	62.11	4.62E7
RHTQENASTIGFVGTHGFS	108.63	1801.8384	17	0.7	901.9271	2	51.98	2.74E8
RDIKPNNMMLDHESIIKI	106.46	1897.9277	16	3.3	949.9743	2	55.17	4.22E7
RHTQENASTIGFVGTHGFSAPELYTDAPHF	105.67	2983.3735	28	2.8	995.4679	3	66.31	6.52E6
RHQALITHKGNPTYLNKD	105.35	1913.9513	16	1.2	957.9841	2	24.54	5.46E7

RGLGIVQEYIYGNDLSNYSPPLNTLDFYLKI	104.93	3305.6494	29	1.5	1102.8921	3	112.67	2.7E7
RVGVVDVQYNGLDFIITRA	104.81	1907.9993	17	4.5	955.0112	2	91.15	1.6E8
KIDGGMGTIFVCNDRI	103.96	1553.6967	14	1.6	777.8568	2	64.00	2.32E8
RAEGEVFINNAPILVNSKI	100.82	1814.9414	17	1.4	908.4792	2	72.17	2.41E7
RILDELNALMKVRS	85.74	1413.8014	12	4.0	707.9108	2	73.70	3.17E6
RSFITFDLSHPEINL	83.64	1631.8195	14	2.4	816.9189	2	94.97	1E8
KFIQAHEENRR	81.30	1142.5469	9	4.4	572.2832	2	11.92	3.57E7
KETLEKHILENRH	77.17	1380.7361	11	2.6	691.3771	2	28.53	1.98E6
KIFDFGLARH	75.52	937.5021	8	2.9	938.5120	1	71.71	3.14E8
NEIASLLQSSLVALPEDRPPIWSIKE	71.28	2661.4741	24	1.9	888.1670	3	94.98	3.9E6
KKITLSVDRV	59.73	930.5498	8	2.2	311.1912	3	24.43	5.86E7

357 aa of Flag-PfkB$_{MP}$

MDYKDDDDKSTIPDRYEFDKKIDGGMGTIFVCNDRILERKVAIKFIQAHEENRRILDELNALMKVRSKHVVQVYDVIED
NERGLGIVQEYIYGNDLSNYSPPLNTLDFYLKIWQIASGISDIHAVNIIHRDIKPNNMMLDHESIIKIFDFGLARHTQE
NASTIGFVGTHGFSAPELYTDAPHFTTAIDVYAFGVTALFLANGTLPSEVLYAYGKPVPNNLFTSLPIANEIASLLQSS
LVALPEDRPPIWSIKETLEKHILENRHQALITHKGNPTYLNKDRKKITLSVDRVGVVDVQYNGLDFIITRAEGEVFINN
APILVNSKIPTSCVLAIGNSQRSNQERSFITFDLSHPEINL

Supplementary Table 7. Mass spectrometry results of the purified protein His-PfkA$_{MP}$(N) during co-purification of His-PfkA$_{MP}$(N) and PfkB$_{MP}$(N)-Flag (refer to **Supplementary Fig.6 middle panel, upper band**, Elution). Peptide fragments identified by mass spectrometry analysis are listed and correspond to the highlights in the His-PfkA$_{MP}$(N) protein sequence below. For the His-PfkA$_{MP}$(N) protein, the -10lgP is 212.91.

Peptide	-10lgP	Mass	Length	ppm	m/z	z	RT	Area
KGLAAAHHAGVVHRD	75.77	1294.7007	13	-1.2	432.5737	3	10.19	7.34E7
KMADEELSDAAKG	72.58	1194.5074	11	0.0	598.2610	2	13.50	2.58E7
KTLDYIKEDDNRY	69.51	1380.6521	11	-0.2	691.3332	2	14.83	2.03E7
RAHDTLLSRKV	67.59	1039.5774	9	0.4	520.7962	2	9.77	7.52E6
RDLKPTNVMISGGYSLNELKI	62.25	2078.0718	19	0.2	693.6980	3	23.80	3.56E6
KITDFGIAKM	56.95	863.4752	8	0.3	432.7450	2	18.26	5.69E7
KVALKTPKN	41.45	755.4905	7	0.1	378.7526	2	9.84	3.78E5
KAVQNILTN	38.40	757.4334	7	0.1	758.4407	1	19.86	9.23E5

293aa of His-PfkA$_{MP}$(N)

MHHHHHHTTSRIGKTIGSRYLIEDLIGEGGMQYVYRAHDTLLSRKVALKTPKNNSATKRFRRSAIVAAKVNHHNVAKTL
DYIKEDDNRYLIEELIEGTDLQNALMKKTKFLDPYLAACVFNQLAKGLAAAHHAGVVHRDLKPTNVMISGGYSLNELKI
TDFGIAKMADEELSDAAKGGSSTLTASQTAVGALPYMAPEAIEVPDKVGPPADIWSIGAMFYHLLTGEFPFSNGLKAVQ
NILTNSITPPPAFLTENPQFRTLASELLDISLSCLKKNPTERPSADDLVKKCELLC

Supplementary Table 8. Mass spectrometry results of the co-purified protein band with His-PfkA$_{MP}$(N) (refer to **Supplementary Fig.6 middle panel, lower band**, Elution). Peptide fragments identified by mass spectrometry analysis are listed and correspond to the highlights in the PfkB$_{MP}$(N)-Flag protein sequence below. For the PfkB$_{MP}$(N)-Flag protein, the -10gP is 285.27.

Peptide	-10lgP	Mass	Length	ppm	m/z	z	RT	Area
RSKHVVQVYDVIEDNERG	83.36	1928.9591	16	0.2	643.9938	3	18.50	2.79E8
KKIDGGMGTIFVCNDRI	76.39	1681.7916	15	1.4	561.6053	3	20.05	3.75E7
KIWQIASGISDIHAVNIIHRD	73.54	2142.1697	19	1.6	715.0650	3	24.05	4.52E7
RDIKPNNMMLDHESIIKI	72.15	1897.9277	16	0.9	633.6505	3	20.23	2.57E7
RRILDELNALMKV	61.50	1314.7329	11	0.5	658.3741	2	25.31	6.95E6
KIFDFGLARH	59.51	937.5021	8	1.0	469.7588	2	25.07	4.16E8

KETLEKHIDYKDDDDK	57.63	1862.8534	15	0.2	621.9586	3	12.17	1.35E7
KFIQAHEENRRI	56.54	1298.6479	10	1.1	433.8904	3	9.47	4.46E8
MSTIPDRYEFDKK	54.63	1369.6514	11	0.7	457.5580	3	16.67	1.09E8
RHTQENASTIGFVGTHGFS	45.68	1801.8384	17	1.6	601.6210	3	20.25	2.85E6
RKVAIKFIQAHEENRRI	31.23	1838.0275	15	0.3	613.6833	3	13.34	2.32E6

259aa of PfkB$_{MP}$(N)-Flag

MSTIPDRYEFDKKIDGGMGTIFVCNDRILERKVAIKFIQAHEENRRILDELNALMKVRSKHVVQVYDVIEDNERGLGIV
QEYIYGNDLSNYSPPLNTLDFYLKIWQIASGISDIHAVNIIHRDIKPNNMMLDHESIIKIFDFGLARHTQENASTIGFV
GTHGFSAPELYTDAPHFTTAIDVYAFGVTALFLANGTLPSEVLYAYGKPVPNNLFTSLPIANEIASLLQSSLVALPEDR
PPIWSIKETLEKHIDYKDDDDK

Supplementary Table 9. Mass spectrometry results of the purified protein His-FHA during co-purification of His-FHA and CSD-Flag (refer to **Supplementary Fig.6 right panel, upper band**, Elution). Peptide fragments identified by mass spectrometry analysis are listed and correspond to the highlights in the His-FHA protein sequence below. For the His-FHA protein, the -10gP is 482.99.

Peptide	-10lgP	Mass	Length	ppm	m/z	z	RT	Area
RAEGEVFINNAPILVNSKIPTSCVLAIGNSQRS	Y	127.74	3311.7183	31	0.9	828.9376	4	26.94
RVGVVDVQYNGLDFIITRA	Y	122.01	1907.0153	17	0.1	954.515	2	27.81
RHQALITHKGNPTYLNKDRK	Y	106.08	2105.113	18	-0.3	527.2854	4	12.23
KGNPTYLNKDRKKITLSVDRV	Y	89.57	2217.2229	19	-2	555.3119	4	14.89
RSFITFDLSHPEINL	Y	41.56	1631.8195	14	6.4	816.9222	2	27.43
MHHHHHHLENRHQALITHKG	Y	34.12	2456.178	19	7.9	492.2467	5	19.77

105aa of His-FHA

MHHHHHHLENRHQALITHKGNPTYLNKDRKKITLSVDRVGVVDVQYNGLDFIITRAEGEVFINNAPILVNSKIPTSCVL
AIGNSQRSNQERSFITFDLSHPEINL

Supplementary Table 10. Mass spectrometry results of the co-purified protein band with His-PfkA$_{MP}$ (refer to **Supplementary Fig.6 right panel, lower band**, Elution). Peptide fragments identified by mass spectrometry analysis are listed and correspond to the highlights in the CSD-Flag protein sequence below. For the CSD-Flag protein, the -10gP is 23.61.

Peptide	-10lgP	Mass	Length	ppm	m/z	z	RT	Area
RAHPVMILDEDYKDDDDK	Y	23.61	2033.8888	17	0.1	678.9703	3	16.29

79aa of CSD-Flag

MYAKNPRYIGNIKNIFYNAYGFIARPLNDDVFFNQSSVYGPRNAKIGDKVCFSMFIGGGAWRAHPVMILDEDYKDDDDK

Supplementary Table 11. Mass spectrometry results of the co-purified protein band with His-PfpC$_{EC039}$ (refer to **Supplementary Fig 10c, the fourth panel**, Elution). Peptide fragments identified by mass spectrometry analysis are listed and correspond to the highlights in the T4 Gp59 protein sequence below. For the Gp59 protein, the -10lgP is 229.88.

Peptide	-10lgP	Mass	Length	ppm	m/z	z	RT	Area
KVEVSAFKEIFEYNPK	89.03	1927.009	16	-0.1	643.3436	3	20.68	5.84E+06
YNWCMR	53.96	928.3684	6	0	465.1915	2	17.83	3.81E+05
KILNIDSQK	53.00	1057.613	9	0.8	529.8143	2	12.53	1.91E+06
AKNVFIETVK	49.61	1147.66	10	-0.4	383.5605	3	14.74	4.38E+05
LYLMIK	38.74	795.4564	6	-0.2	398.7354	2	17.91	1.09E+06
RDKYFFQK	36.54	1130.587	8	-0.4	377.8696	3	13.96	3.83E+05

VQSSYIFK	33.43	970.5123	8	-0.4	971.5192	1	16.83	3.64E+05
FKFEEDIRNIYYFSK	30.79	1997.989	15	0.7	667.004	3	22.64	1.45E+06

Escherichia phage T4 Gp59

MIKLRMPAGGERYIDGKSVYKLYLMIKQHMNGKYDVIKYNWCMRVSDAAYQKRRDKYFFQKLSEKYKLKELALIFISNL
VANQDAWIGDISDADALVFYREYIGRLKQIKFKFEEDIRNIYYFSKKVEVSAFKEIFEYNPKVQSSYIFKLLQSNIISF
ETFILLDSFLNIIDKHDEQTDNLVWNNYSIKLKAYRKILNIDSQKAKNVFIETVKSCKY

Supplementary Table 12. Bacterial strains and plasmids used in this study. R indicates resistance, Gm indicates gentamycin, Amp indicates ampicillin, MP indicates MPAO1 strain. KKP indicates the *pfkA-pfkB-pfpC* cascade.

	Description	Source
**Strains**		
PAO1	wild-type	[2]
MPAO1	wild-type	[3]
MP-ΔPf4	whole Pf4 prophage removed from MPAO1 host chromosome	[3]
MP-ΔPf6core	core genes from MP-4976 to MP_4987 removed from MPAO1 host chromosome	this study
MP-ΔPf4ΔPf6core	whole Pf4 prophage removed from MP-ΔPf6core host chromosome	this study
MP-ΔPf4ΔPf6	whole Pf6 prophage removed from MP-ΔPf4 host chromosome	this study
Δ*pfkA*	*pfkA* gene removed from the MPAO1 host chromosome	this study
ΔKK$_{MP}$	*pfkA* and *pfkB* genes removed from the MPAO1 host chromosome	this study
ΔKKP$_{MP}$	*pfkA, pfkB* and *pfpC* genes removed from the MPAO1 host chromosome	this study
pVIII::GFP	*gVIII* gene encoding Pf4 major coat protein pVIII in-frame fused with *gfp* gene in PAO1 host chromosome	this study
MP-pVIII::GFP	*gVIII* gene encoding Pf4 major coat protein pVIII in-frame fused with *gfp* gene in MPAO1 host chromosome	this study
MP-Δ*mvaU*Δ*mvaT*	*mvaT* and *mvaU* double deletion mutant derived from MPAO1	[3]
MP::*mvaU*^{67D}::Gm	*mvaU* gene in MPAO1 chromosome replaced with *mvaU*^{67D} followed by Gm resistance cassette with its own promoter	this study
MP::*mvaU*-His	*mvaU* gene in MPAO1 chromosome fused with His-tag at its C-terminus to express MvaU-His	this study
PAO1::*pilC*$_{MP}$	*pilC* gene from MPAO1 integrated into Δ*pilC* host chromosome	this study
PAO1::*pilC*$_{MP}$::KKP$_{MP}$	KKP$_{MP}$ cassette integrated into PAO1::*pilC*$_{MP}$ host chromosome	this study
PAO1::*pilC*$_{MP}$::K^{GFI}KP$_{MP}$	mutant KKP$_{MP}$ cascade (the 307th -309th aa codon GGATTCATT in *pfkA* was mutated to GACTATGCA) integrated into PAO1::*pilC*$_{MP}$ host chromosome	this study
WM3064	*thrB*1004 *pro thi rpsL hsdS lacZ*ΔM15 RP4-1360) Δ(*araBAD*)567 Δ*dapA*1341::[*erm pir(*wt)]	W. Metcalf, UIUC
**Plasmids**		
pEX18Ap	Ap^{R}, *oriT*$^{+}$, *sacB*$^{+}$, gene replacement vector	[4]
pEX18Gm	Gm^{R}, *oriT*$^{+}$, *sacB*$^{+}$, gene replacement vector	[4]
pFLP2	Ap^{R}, Flp recombinase-expressing plasmid	[4]
pPS856	Ap^{R}, Gm^{R}; for amplifying gentamycin resistance cassette	[4]
pEX18Ap-Pf4-up-Gm-down	Gm^{R}, Ap^{R}, for deleting Pf4	[3]
pEX18Gm-ΔPf6core-up-down	Gm^{R}, Ap^{R}, for deleting Pf6core region	this study
pEX18Gm-up-pVIII::GFP-down	Gm^{R}, Ap^{R}, for Pf4 *gVIII* in-frame fused with *gfp* gene in MPAO1 host chromosome	this study
pEX18Ap-*mvaU*-up-Gm-down	Gm^{R}, Ap^{R}, for deleting *mvaU*	[3]

pEX18Ap-*mvaT*-up-Gm-down	Gm^R, Ap^R, for deleting *mvaT*	[3]
pEX18Gm-*pfkA*-up-down	Gm^R, Ap^R, for deleting *pfkA* in MPAO1 host chromosome	this study
pEX18Gm-KK_{MP}-up-down	Gm^R, Ap^R, for deleting KK_{MP} in MPAO1 host chromosome	this study
pEX18Gm-KKP_{MP} -up-down	Gm^R, Ap^R, for deleting KKP_{MP} in MPAO1 host chromosome	this study
pEX18Gm-Pf6-up-down	Gm^R, Ap^R, for deleting Pf6 in MPAO1 host chromosome	this study
pMD19	Ap^R, for cloning of *pfiA* and *intF6* genes	Takara
pMD19-*pfiA*	Ap^R, *pfiA* in Pf4 was cloned into T-vector pMD19	this study
pMD19-*intF6*	Ap^R, *intF6* in Pf6 was cloned into T-vector pMD19	this study
pHERD20T	Ap^R, expression vector with araC-P_{BAD} promoter	[5]
pHERD20T-*mvaU*	Ap^R, *mvaU* in pHERD20T NcoI/HindIII	this study
pHERD20T-$mvaU^{S26A}$	Ap^R, the 26th aa codon AGC in *mvaU* was mutated to GCC in pHERD20T-*mvaU*	this study
pHERD20T-$mvaU^{S26D}$	Ap^R, the 26th aa codon AGC in *mvaU* was mutated to GAC in pHERD20T-*mvaU*	this study
pHERD20T-$mvaU^{T50A}$	Ap^R, the 50th aa codon ACC in *mvaU* was mutated to GCC in pHERD20T-*mvaU*	this study
pHERD20T-$mvaU^{T50D}$	Ap^R, the 50th aa codon ACC in *mvaU* was mutated to GAC in pHERD20T-*mvaU*	this study
pHERD20T-$mvaU^{T65A}$	Ap^R, the 65th aa codon ACC in *mvaU* was mutated to GCC in pHERD20T-*mvaU*	this study
pHERD20T-$mvaU^{T65D}$	Ap^R, the 65th aa codon ACC in *mvaU* was mutated to GAC in pHERD20T-*mvaU*	this study
pHERD20T-$mvaU^{S67A}$	Ap^R, the 67th aa codon AGC in *mvaU* was mutated to GCC in pHERD20T-*mvaU*	this study
pHERD20T-$mvaU^{S67D}$	Ap^R, the 67th aa codon AGC in *mvaU* was mutated to GAC in pHERD20T-*mvaU*	this study
pHERD20T-$mvaU^{S67A}$-$mvaU^{S67D}$	Ap^R, $mvaU^{S67A}$ and $mvaU^{S67D}$ were co-expressed in pHERD20T plasmid	this study
pHERD20T-$mvaU^{S108A}$	Ap^R, the 108th aa codon TCC in *mvaU* was mutated to GCC in pHERD20T-*mvaU*	this study
pHERD20T-$mvaU^{S108D}$	Ap^R, the 108th aa codon TCC in *mvaU* was mutated to GAC in pHERD20T-*mvaU*	this study
pHERD20T-$mvaU^{S113A}$	Ap^R, the 113th aa codon TCC in *mvaU* was mutated to GCC in pHERD20T-*mvaU*	this study
pHERD20T-$mvaU^{S113D}$	Ap^R, the 113th aa codon TCC in *mvaU* was mutated to GAC in pHERD20T-*mvaU*	this study
pHERD20T-*mvaU*-His-*pfkA*	Ap^R, N terminal His tagged *mvaU* was coexpressed with *pfkA* in pHERD20T NcoI/HindIII	this study
pHERD20T-*mvaU*-His-*pfkB*	Ap^R, N terminal His tagged *mvaU* was coexpressed with *pfkB* in pHERD20T NcoI/HindIII	this study
pHERD20T-*mvaU*-His-*pfkA*-*pfkB*	Ap^R, N terminal His tagged *mvaU* was coexpressed with *pfkA-pfkB* in pHERD20T NcoI/HindIII	this study
pHERD20T-*mvaU*-His-KKP	Ap^R, N terminal His tagged *mvaU* was coexpressed with KKP in pHERD20T NcoI/HindIII	this study
pHERD20T-*pfkA*	Ap^R, *pfkA* in pHERD20T NcoI/HindIII	this study
pHERD20T-$pfkA^{CSD}$	Ap^R, mutant *pfkA* lacking CSD domain in pHERD20T NcoI/HindIII	this study
pHERD20T-*CSD*	Ap^R, CSD domain of *pfkA* in pHERD20T NcoI/HindIII	this study
pHERD20T-*pfkB*	Ap^R, *pfkB* in pHERD20T NcoI/HindIII	this study
pHERD20T-$pfkB^{FHA}$	Ap^R, mutant *pfkB* lacking FHA domain in pHERD20T NcoI/HindIII	this study
pHERD20T-*FHA*	Ap^R, FHA domain of *pfkB* in pHERD20T NcoI/HindIII	this study

pHERD20T-*pfpC*	Ap^{R}, *pfpc* in pHERD20T NcoI/HindIII	this study
pHERD20T-*pfkA-pfkB*	Ap^{R}, *pfkA-pfkB* in pHERD20T NcoI/HindIII	this study
pHERD20T-*pfkA*^{CSD}-*pfkB*	Ap^{R}, mutant *pfkA-pfkB* without CSD domain in pHERD20T NcoI/HindIII	this study
pHERD20T-*pfkA-pfkB*^{FHA}	Ap^{R}, mutant *pfkA-pfkB* without FHA domain in pHERD20T NcoI/HindIII	this study
pHERD20T-*pfkA*^{CSD}-*pfkB*^{FHA}	Ap^{R}, mutant *pfkA-pfkB* without both CSD and FHA domains in pHERD20T NcoI/HindIII	this study
pHERD20T-*pfkA*^{GGM}-*pfkB*	Ap^{R}, mutant GGM motif in PfkA in *pfkA-pfkB* in pHERD20T NcoI/HindIII	this study
pHERD20T-*pfkA*^{HRD}-*pfkB*	Ap^{R}, mutant HRD motif in PfkA in *pfkA-pfkB* in pHERD20T NcoI/HindIII	this study
pHERD20T-*pfkA*^{DFG}-*pfkB*	Ap^{R}, mutant DFG motif in PfkA in *pfkA-pfkB* in pHERD20T NcoI/HindIII	this study
pHERD20T-*pfkA*^{GFI}-*pfkB*	Ap^{R}, mutant GFI motif in PfkA in *pfkA-pfkB* in pHERD20T NcoI/HindIII	this study
pHERD20T-*pfkA-pfkB*^{GGM}	Ap^{R}, mutant GGM motif in PfkB in *pfkA-pfkB* in pHERD20T NcoI/HindIII	this study
pHERD20T-*pfkA-pfkB*^{HRD}	Ap^{R}, mutant HRD motif in PfkB in *pfkA-pfkB* in pHERD20T NcoI/HindIII	this study
pHERD20T-*pfkA-pfkB*^{DFG}	Ap^{R}, mutant DFG motif in PfkB in *pfkA-pfkB* in pHERD20T NcoI/HindIII	this study
pHERD20T-*pfkA*^{GGM}-*pfkB*^{GGM}	Ap^{R}, mutant GGM motifs in both PfkA and PfkB in *pfkA-pfkB* in pHERD20T NcoI/HindIII	this study
pHERD20T-*pfkA*^{HRD}-*pfkB*^{HRD}	Ap^{R}, mutant HRD motifs in both PfkA and PfkB in *pfkA-pfkB* in pHERD20T NcoI/HindIII	this study
pHERD20T-*pfkA*^{DFG}-*pfkB*^{DFG}	Ap^{R}, mutant DFG motifs in both PfkA and PfkB in *pfkA-pfkB* in pHERD20T NcoI/HindIII	this study
pHERD20T-*pfkA*$^{GGM+HRD+DFG}$-*pfkB*	Ap^{R}, the GGM, HRD and DFG motif in PfkA were mutated as PAO1::MP-*pilC*::K$^{GGM+HRD+DFG}$K$^{GGM+HRD+DFG}$P in *pfkA-pfkB,* and the generated *pfkA*$^{GGM+HRD+DFG}$-*pfkB* was ligated to pHERD20T NcoI/HindIII	this study
pHERD20T-*pfkA-pfkB*$^{GGM+HRD+DFG}$	Ap^{R}, the GGM, HRD and DFG motif in PfkB were mutated as PAO1::MP-*pilC*::K$^{GGM+HRD+DFG}$K$^{GGM+HRD+DFG}$P in *pfkA-pfkB,* and the generated *pfkA*$^{GGM+HRD+DFG}$-*pfkB* was ligated to pHERD20T NcoI/HindIII	this study
pHERD20T-*pfkA*$^{GGM+HRD+DFG}$ -*pfkB*$^{GGM+HRD+DFG}$	Ap^{R}, the GGM, HRD and DFG motif in both PfkA and PfkB were mutated as PAO1::MP-*pilC*::K$^{GGM+HRD+DFG}$K$^{GGM+HRD+DFG}$P in *pfkA-pfkB,* and the generated *pfkA*$^{GGM+HRD+DFG}$-*pfkB*$^{GGM+HRD+DFG}$ was ligated to pHERD20T NcoI/HindIII	this study
pHERD20T-*pfkA-pfpC*	Ap^{R}, *pfkA-pfpC* in pHERD20T NcoI/HindIII	this study
pHERD20T-*pfkB-pfpC*	Ap^{R}, *pfkB-pfpC* in pHERD20T NcoI/HindIII	this study
pHERD20T-*pfkA-pfkB-pfpC*	Ap^{R}, KKP$_{MP}$ (*pfkA-pfkB-pfpC*) in pHERD20T NcoI/HindIII	this study
pHERD20T-*pfkA*^{GGM}-*pfkB*	Ap^{R}, the 23th-25th aa codon GGAGGCATG in *pfkA* was mutated to GCAGCGTGT in pHERD20T-*pfkA-pfkB*	this study
pHERD20T-*pfkA*^{HRD}-*pfkB*	Ap^{R}, the 131th-133th aa codon CATCGCGAC in *pfkA* was mutated to TTCGAAAAT in pHERD20T-*pfkA-pfkB*	this study
pHERD20T-*pfkA*^{DFG}-*pfkB*	Ap^{R}, the 154th-157th aa codon GACTTCGGT in *pfkA* was mutated to AATTATGCA in pHERD20T-*pfkA-pfkB*	this study
pHERD20T-*pfkA-pfkB*^{GGM}	Ap^{R}, the 16th-18th aa codon GGCGGCATG in *pfkB* was mutated to GCAGCGTGT in pHERD20T-*pfkA-pfkB*	this study
pHERD20T-*pfkA-pfkB*^{HRD}	Ap^{R}, the 121th-123th aa codon CATAGAGAT in *pfkB* was mutated to TTCGAAAAT in pHERD20T-*pfkA-pfkB*	this study
pHERD20T-*pfkA-pfkB*^{DFG}	Ap^{R}, the 141th-143th aa codon GACTTTGGC in *pfkB* was mutated to AATTATGCA in pHERD20T-*pfkA-pfkB*	this study
pHERD20T-*2208*	Ap^{R}, *2208* in pHERD20T NcoI/HindIII	this study
pHERD20T-*2209*	Ap^{R}, *2209* in pHERD20T NcoI/HindIII	this study

pHERD20T-*2210*	Ap^{R}, *2210* in pHERD20T NcoI/HindIII	this study
pHERD20T-*2208-2209*	Ap^{R}, *2208-2209* in pHERD20T NcoI/HindIII	this study
pHERD20T-*2208-2210*	Ap^{R}, *2208-2210* in pHERD20T NcoI/HindIII	this study
pHERD20T-*2209-2210*	Ap^{R}, *2209-2210* in pHERD20T NcoI/HindIII	this study
pHERD20T-*2208-2209-2210*	Ap^{R}, *2208-2209-2210* (KKP$_{SW}$ in marine *Shewanella* W3-18-1) in pHERD20T NcoI/HindIII	this study
pHERD20T-K^{GFA}KP	Ap^{R}, *2208-2209-2210* ^{GFA} (K^{GFA}KP$_{SW}$ with GFA mutation in marine *Shewanella* W3-18-1) in pHERD20T NcoI/HindIII	this study
pHERD20T-K$^{HRD+DFG}$K$^{HRD+DFG}$P	Ap^{R}, *2208-2209*$^{HRD+DFG}$-*2210*$^{HRD+DFG}$ (K^{GFA}KP$_{SW}$ with GFA mutation in marine *Shewanella* W3-18-1) in pHERD20T NcoI/HindIII	this study
pHERD20T-*PA0717*	Ap^{R}, *PA0717* in pHERD20T NcoI/HindIII	[6]
pHERD20T-*PA0718*	Ap^{R}, *PA0718* in pHERD20T NcoI/HindIII	[6]
pHERD20T-*PA0719*	Ap^{R}, *PA0719* in pHERD20T NcoI/HindIII	[6]
pHERD20T-*PA0720*	Ap^{R}, *PA0720* in pHERD20T NcoI/HindIII	[6]
pHERD20T-*PA0721*	Ap^{R}, *PA0721* in pHERD20T NcoI/HindIII	[6]
pHERD20T-*PA0722*	Ap^{R}, *PA0722* in pHERD20T NcoI/HindIII	[6]
pHERD20T-*PA0723*	Ap^{R}, *PA0723*in pHERD20T NcoI/HindIII	[6]
pHERD20T-*PA0724*	Ap^{R}, *PA0724* in pHERD20T NcoI/HindIII	[6]
pHERD20T-*PA0725*	Ap^{R}, *PA0725* in pHERD20T NcoI/HindIII	[6]
pHERD20T-*PA0726*	Ap^{R}, *PA0726* in pHERD20T NcoI/HindIII	[6]
pHERD20T-*PA0726.1*	Ap^{R}, *PA0726.1* in pHERD20T NcoI/HindIII	[6]
pHERD20T-*repF4*	Ap^{R}, *repF4* in pHERD20T NcoI/HindIII	[6]
pHERD20T-*intF4*	Ap^{R}, *intF4* in pHERD20T NcoI/HindIII	[6]
pHERD20T-*pfiA*	Ap^{R}, *pfiA* in pHERD20T NcoI/HindIII	[6,7]
pET28b	Km^{R}, expression vector with T7 promoter	Novagen
pET28b-*pfkA*-His	Km^{R}, lacI^{q}, pET28b P$_{T7–lac}$:: *pfkA*$_{MP}$-His with PfkA C-terminus His-tagged	this study
pET28b-*pfkB*-His	Km^{R}, lacI^{q}, pET28b P$_{T7–lac}$:: *pfkB*$_{MP}$-His with PfkB C-terminus His-tagged	this study
pET28b-*pfkB*$_{MP}$-*pfkA*$_{MP}$-His	Km^{R}, lacI^{q}, pET28b P$_{T7–lac}$::*pfkB*$_{MP}$-*pfkA*$_{MP}$-His with PfkA C-terminus His-tagged	this study
pET28b-*pfpC*$_{MP}$-*pfkB*$_{MP}$-*pfkA*$_{MP}$-His	Km^{R}, lacI^{q}, pET28b P$_{T7–lac}$::*pfpC*$_{MP}$-*pfkB*$_{MP}$-*pfkA*$_{MP}$-His with PfkA C-terminus His-tagged	this study
pET28b-His-*pfkB*$_{MP}$-*pfkA*$_{MP}$-Flag	Km^{R}, lacI^{q}, pET28b P$_{T7–lac}$::His-*pfkB*$_{MP}$-*pfkA*$_{MP}$-Flag with PfkB N-terminus His-tagged and PfkA C-terminus Flag-tagged	this study
pET28b-His-*pfkA*$_{MP}$(N)	Km^{R}, lacI^{q}, pET28b P$_{T7–lac}$::His-*pfkA*$_{MP}$(N) with PfkA N-terminus kinase domain His-tagged	this study
pET28b-His-*FHA*	Km^{R}, lacI^{q}, pET28b P$_{T7–lac}$::His-*FHA* with PfkB C-terminus FHA domain His-tagged	this study
pET21a	Amp^{R}, expression vector with T7 promoter	Novagen
pET21a-*pfkB*$_{MP}$(N)-Flag	Amp^{R}, pET21a P$_{T7–lac}$::*pfkB*$_{MP}$(N) with PfkB N-terminus kinase domain Flag-tagged	this study
pET21a-*CSD*-Flag	Amp^{R}, pET21a P$_{T7–lac}$::*CSD* with PfkA C-terminus CSD domain Flag-tagged	this study

pETduet	Amp^R, expression vector	lab strored
pETduet-His-*pfpC*$_{EC039}$-*gp59*	Ap^R, His-*pfpC*$_{EC039}$ in pETduet NcoI/HindIII, *gp59* in pETduet NdeI/XhoI	this study
pETduet-His-*pfpC*$_{EC039}$-*gp59*^{G32E}	Ap^R, His-*pfpC*$_{EC039}$ in pETduet NcoI/HindIII, *gp59*^{G32E} in pETduet NdeI/XhoI	this study
pET28a-Tev	Km^R, expression vector with T7 promoter	lab strored
pET28a-Tev-*gp59*	Km^R, $lacI^q$, pET28a-Tev P_{T7-lac}::His-Tev-*gp59* with GP59 N-terminus His-Tev-tagged	this study
pET28a-Tev-*gp59*^{G32E}	Km^R, $lacI^q$, pET28a-Tev P_{T7-lac}::His-Tev *gp59*^{G32E} with $GP59^{G32E}$ N-terminus His-Tev-tagged	this study
pTac	Cm^R, P_{T7} expression vector	lab stored
pTac-*pfkA*	Cm^R, *pfkA* in pTac EcoRI/BamHI	this study
pTac-*pfkB*	Cm^R, *pfkB* in pTac EcoRI/BamHI	this study
pTac-KK_{MP}	Cm^R, KK_{MP} (*pfkAB*) in pTac EcoRI/BamHI	this study
pTac-KKP_{MP}	Cm^R, KKP_{MP} in pTac EcoRI/BamHI	this study
pTac-$K^{GFI}KP_{MP}$	Cm^R, $K^{GFI}KP_{MP}$ in pTac EcoRI/BamHI	this study
pTac-KKP_{SW}	Cm^R, KKP_{SW} in pTac EcoRI/BamHI	this study
pTac-KKP_{Ec039}	Cm^R, KKP_{Ec039} in pTac EcoRI/BamHI	this study

Supplementary Table. 13 Oligonucleotides used for gene knockout and DNA sequencing. F indicates forward primer and R indicates reverse primer. The red letters indicate the mutation of nucleotides.

Primer name	Sequence (5'-3')	Purpose
**Primers used for construction of MP-ΔPf6core and MP-ΔPf4ΔPf6core**		
Pf6core-up-F	ACGACGGCCAGTGCCAAGCTTTCTAAGTTCACATTCATGCACTCTTTC	
Pf6core-up-R	TGAGCGGGTCTCATTAGTCACCGTCGTAATCCCCC	Construction of
Pf6core-down-F	TGACTAATGAGACCCGCTCAATGGACA	pEX18Gm-ΔPf6core-up-down
Pf6core-down-R	GGTACCCGGGGATCCTCTAGAGAGTGGTGATAGCGAAACTCGAT	
Pf6core-conf-SF	TGAATCATCGCTACGCTCCTC	
Pf6core-conf-SR	ACGGCTGCTTGGTTGGTCT	Verification of Pf6core knockout in
Pf6core-conf-LF	CATGGCAGACTCTATCAGCCA	MP-ΔPf6core and MP-ΔPf4ΔPf6core
Pf6core-conf-LR	ATCGCATATCCCTTGCGCAGATA	
Pf4-conf-SF	ATCTTTCCCGGGCTTTACG	
Pf4-conf-SR	GCAATTGGCAAAGTGTTCGA	Verification of Pf4 knockout in
Pf4-conf-LF	TACGAGGCTGTTGAGGAGTTA	MP-ΔPf4ΔPf6core, and ΔPf4
Pf4-conf-LR	CCGTGCCGAGGTAGTGATGTC	
**Primers used for construction of pVIII::GFP and MP-pVIII::GFP**		
pVIII-gfp-up-F	GCCAGTGCCAAGCTTGCATGCAGGACAAACAAGACAAGACCCCG	
pVIII-gfp-up-R	TTCTCCTTTACTCGCCTTGCGCAACATGCT	
pVIII-gfp-F2	GCAAGGCGAGTAAAGGAGAAGAACTTTTCACTGGA	Construction of
pVIII-gfp-R2	CCAGAGCACCCGTTATTTGTATAGTTCATCCATGCCATG	pEX18Gm-pVIII::GFP-up-down
pVIII-gfp-down-F	ACAAATAACGGGTGCTCTGGTCGGTG	
pVIII-gfp-down-R	TATGACCATGATTACGAATTCCGGTGCCCTTGAGGATGTAA	
pVIII-gfp-conf-SF	GCGAATACAACATCGAGCCG	
pVIII-gfp-conf-SR	GGTCTGGGAGAAGGTGTAGGAAT	Verification of strains pVIII::GFP and MP-pVIII::GFP
pVIII-gfp-conf-LF	ATGAACATGTTTGCAACCCAA	
pVIII-gfp-conf-LR	TCAGTCCTTCAGCAGAATGAG	
**Primers used for construction of PAO1::*pilC*$_{MP}$::KKP$_{MP}$**		
KKP$_{MP}$-F1	ACGACGGCCAGTGCCAAGCTTTAGTTGATTACCACTGAGGAGGTGG	Amplification of the 697 bp
KKP$_{MP}$-R1	CGACCCAAGTACCGCCACCTAAATGGCACTCTAATAAATAAAATTAAATTTATCGCTTC	downstream of *pfkA*
KKP$_{MP}$-F2	GAAGCGATAAATTTAATTTTATTTATTAGAGTGCCATTTAGGTGGCGGTACTTGGGTCG	Amplification of Gm resistance gene

KKP$_{MP}$-R2	AGCACATCCTGTTATGATCCTTGATGAGTAGAGTTCCTATACTTTCTAGAGAATAGGAA	with its 303 bp promoter
KKP$_{MP}$-F3	TTCCTATTCTCTAGAAAGTATAGGAACTCTACTCATCAAGGATCATAACAGGATGTGCT	Amplification of KKP cascades and
KKP$_{MP}$-R3	TGTCCCAGGTTCAAGTCCCGGTGTAGCCACCATGAGAATCAATACAGAGCAGAGCTAGG	upstream 404 bp promoter the start
		codon of *pf6r* was mutated to TGA)
KKP$_{MP}$-F4	CCTAGCTCTGCTCTGTATTGATTCTCATGGTGGCTACACCGGGACTTGAACCTGGGACA	Amplification of the 836 bp from Pf6
KKP$_{MP}$-R4	TATGACCATGATTACGAATTCGGGCAAGCCGGCGCGTTC	*attR* to upstream
**Primers used for construction of Δ*pfkA***		
pfkA-F1-F	ACGACGGCCAGTGCCAAGCTT CGCGGGCTTGGCATCGTACAAGAA	Construction of
pfkA-F1-R	ATTAGAGTGCCAT AAGTTTATCTCCGGATGAGATAGGT	pEX18Gm-*pfkA*-up-down
pfkA-F2-F	CGGAGATAAACTT ATGGCACTCTAATAAATAAAATTA	
pfkA-F2-R	TATGACCATGATTACGAATTC ACAGTCGCTACGCGTAAGCT	
pfkA-LF	ATTGATGGCGGCATGGGTAC	Verification of strain Δ*pfkA*
pfkA-LR	TGGTAATCTGTAGTGAAGTTA	
**Primers used for construction of ΔKK$_{MP}$**		
KK$_{MP}$-F1-F	ACGACGGCCAGTGCCAAGCTT GGCAAATGACTGGCTGATAGAGTC	
KK$_{MP}$ -F1-R	TAATTTTATTTATTAGAGTGCCATTCCTAGTCATCCTTGATGGGGAAAG	Construction of
KK$_{MP}$ -F2-F	CTTTCCCCATCAAGGATGACTAGGAATGGCACTCTAATAAATAAAATTA	pEX18Gm-KK$_{MP}$-up-down
KK$_{MP}$ -F2-R	TATGACCATGATTACGAATTC ACAGTCGCTACGCGTAAGCT	
KK$_{MP}$ -LF	GAAGAGTCGCTGGAATGAATGC	Verification of strain ΔKK$_{MP}$
KK$_{MP}$ -LR	TGGTAATCTGTAGTGAAGTTA	
**Primers used for construction of ΔKKP$_{MP}$**		
KKP$_{MP}$-F1-F	ACGACGGCCAGTGCCAAGCTTTACGAGGTACTTAGTCACCGTCGT	Construction of pEX18Gm-KKP$_{MP}$-up-down
KKP$_{MP}$-F1-R	ATTAGAGTGCCATGCACTCTTTCTCAGCTTGGGGGGAT	
KKP$_{MP}$-F2-F	CTGAGAAAGAGTGC ATGGCACTCTAATAAATAAAATTA	
KKP$_{MP}$-F2-R	TATGACCATGATTACGAATTC ACAGTCGCTACGCGTAAGCT	
KKP$_{MP}$ -LF	CATGCCCTCACCCCACGGATGCGATACACC	Verification of strain ΔKKP$_{MP}$
KKP$_{MP}$ -LR	TGGTAATCTGTAGTGAAGTTA	
**Primers used for Pf6 deletion**		
Pf6-F1-F	ACGACGGCCAGTGCCAAGCTTTCTGGCTACAAATTGACCACATG	Construction of pEX18Gm-Pf6-up-down
Pf6-F1-R	AGCGCAGCATTGTTTTGGTCGGCAGACTACAA	
Pf6-F2-F	GACCAAAACAATGCTGCGCTCTAACCGACT	

Pf6-F2-R	GGTACCCGGGGATCCTCTAGAGGGCAAGCCGGCGCGTTC	
Pf6-LF	ACCAGTGGCGTGTCTGTTTC	Verification of strain Pf6 deletion strains
Pf6-LR	GGAAGGCAAGGACTACATCGT	

Primers used for construction of MPAO1::*mvaU*-His

mvaU-His -F1-F	ACGACGGCCAGTGCCAAGCTT ATCTGGCAACAACTCAAGCC	Construction of pEX18Gm- *mvaU*-His -up-down
mvaU-His -F1-R	CAGGATGCCGTCGGGAAAACCGGCTTAGTGATGATGATGATGATGGCGTTGCAGCCAGG	
mvaU-His -F2-F	CCTGGCTGCAACGCCATCATCATCATCATCACTAAGCCGGTTTTCCCGACGGCATCCTG	
mvaU-His -F2-R	TATGACCATGATTACGAATTC AGGCCCAGGTTCGCGGGCAGTT	
mvaU-His -LF	AATTCACCTTCGAATCCGCCCA	Verification of strain MPAO1:: *mvaU*-His
mvaU-His -LR	GTTGTGCATCTGGTCATGCT	

Primers used for construction of MPAO1::*mvaU*^{S67D}:::Gm

mvaU^{S67D}-F1-F	ACGACGGCCAGTGCCAAGCTTATCTGGCAACAACTCAAGCC	Construction of pEX18Gm- *mvaU*^{S67D}:::Gm-up-down
mvaU^{S67D}-F1-R	AACTCGGCAAGTTTGGACATTCGTTTCACTCCCGTTCTAA	
mvaU^{S67D}-F2-F	TTAGAACGGGAGTGAAACGAATGTCCAAACTTGCCGAGTT	
mvaU^{S67D}-F2-R	TTTTCTTGTTATCGCAATAGTTAGCGTTGCAGCCAGGATT	
Gm-F	AATCCTGGCTGCAACGCTAACTATTGCGATAACAAGAAAA	
Gm-R	ATGCCGTCGGGAAAACCGGCTTAGGTGGCGGTACTTGGGT	
mvaU^{S67D} -F3-F	ACCCAAGTACCGCCACCTAAGCCGGTTTTCCCGACGGCAT	
mvaU^{S67D} -F3-R	TATGACCATGATTACGAATTC AGGCCCAGGTTCGCGGGCAGTT	
mvaU^{S67D} -LF	AATTCACCTTCGAATCCGCCCA	Verification of strain MPAO1 *mvaU*^{S67D}:::Gm
mvaU^{S67D} -LR	GTTGTGCATCTGGTCATGCT	

Primers used for construction of pHERD20T-*mvaU* and related mutants

pHERD20T-*mvaU*-F	AAGAAGGAGATATACATACCATGTCCAAACTTGCCGAGTTC	
pHERD20T-*mvaU*-R	CGACGGCCAGTGCCAAGCTTTTAGCGTTGCAGCCAGGATTC	
pHERD20T-*mvaU*^{S26A}-F	GCTGAAAGCCGACAGCAGCCTGAAGCAGGAACTGGAAT	
pHERD20T-*mvaU*^{S26A}-R	GCTGCTGTCGGCTTTCAGCTTTTCCAGCAGGGCCAGTT	
pHERD20T-*mvaU*^{S26D}-F	AAGCTGAAAGACGACAGCAGCCTGAAGCAGGAACTGGAAT	
pHERD20T-*mvaU*^{S26D}-R	CAGGCTGCTGTCGTCTTTCAGCTTTTCCAGCAGGGCCAGT	
pHERD20T-*mvaU*^{T50A}-F	TACGGCATGGCCCTGCACAACATCATCGCCATCCTCGACC	Construction of pHERD20T-*mvaU* and related single site mutations in MvaU
pHERD20T-*mvaU*^{T50A}-R	GTTGTGCAGGGCCATGCCGTACTTGTCCATCAACGCCTGC	

pHERD20T-*mvaU*^{T50D}-F	TACGGCATGGACCTGCACAACATCATCGCCATCCTCGACC	
pHERD20T-*mvaU*^{T50D}-R	GTTGTGCAGGTCCATGCCGTACTTGTCCATCAACGCCTGC	
pHERD20T-*mvaU*^{T65A}-F	AAGGCTCCGGTCGCCGTCAGCGCCGCTCCGCAGCGCCGTGCCCGC	
pHERD20T-*mvaU*^{T65A}-R	GGCGCTGACGGCGACCGGAGCCTTGGGGTCGAGGATGGCGATGAT	
pHERD20T-*mvaU*^{T65D}-F	GCTCCGGTCGACGTCAGCGCCGCTCCGCAGCGCCGTGCC	
pHERD20T-*mvaU*^{T65D}-R	GCTGACGTCGACCGGAGCCTTGGGGTCGAGGATGGCGAT	
pHERD20T-*mvaU*^{S67A}-F	AAGGCTCCGGTCACCGTCGCCGCCGCTCCGCAGCGCCGTGCCCGC	
pHERD20T-*mvaU*^{S67A}-R	GGCGGCGACGGTGACCGGAGCCTTGGGGTCGAGGATGGCGATGAT	
pHERD20T-*mvaU*^{S67D}-F	GCTCCGGTCACCGTCGACGCCGCTCCGCAGCGCCGTGCC	
pHERD20T-*mvaU*^{S67D}-R	GGAGCGGCGTCGACGGTGACCGGAGCCTTGGGGTCGAGGATGGCGAT	
pHERD20T-*mvaU*^{S67A} *mvaU*^{S67D} -F1-F	AGGAGATATACATACCC ATGTCCAAACTTGCCGAGTT	
pHERD20T-*mvaU*^{S67A} *mvaU*^{S67D} -F1-R	AACCCATGGGAGAGAGACGGCAGAACGGGCCCGTTTTAGCGTTGCAGCCAGGATTCGAC	
pHERD20T-*mvaU*^{S67A} *mvaU*^{S67D} -F2-F	GTCGAATCCTGGCTGCAACGCTAAAACGGGCCCGTTCTGCCGTCTCTCTCCCATGGGTT	
pHERD20T-*mvaU*^{S67A} *mvaU*^{S67D} -F2-R	ACGACGGCCAGTGCCAAGCTT TTAGCGTTGCAGCCAGGATTCGA	
pHERD20T-*mvaU*^{S108A}-R	CGACGGCCAGTGCCAAGCTTTTAGCGTTGCAGCCAGGATTCGACGGTTTCGGAACCGGCCTGTTCTTTC	
pHERD20T-*mvaU*^{S108D}-R	CGACGGCCAGTGCCAAGCTTTTAGCGTTGCAGCCAGGATTCGACGGTTTCGGAACCGTCCTGTTCTTTC	
pHERD20T-*mvaU*^{S113A}-R	CGACGGCCAGTGCCAAGCTTTTAGCGTTGCAGCCAGGCTTCGACGGTTTC	
pHERD20T-*mvaU*^{S113D}-R	CGACGGCCAGTGCCAAGCTTTTAGCGTTGCAGCCAGTCTTCGACGGTTTC	

Primers used for cloning KKP$_{MP}$ components and domains of PfkA and PfkB

pHERD20T-*pfkA*-F	AGGAGATATACATACCCATGACGACTTCGAGAATCGGAAAGAC	
pHERD20T-*pfkA*-R	ACGACGGCCAGTGCCAAGCTTCTACTCATCAAGGATCATAACAGGATG	
pTac--*pfkA*-F	CAATTTCACACAGGAGAATTCATGACGACTTCGAGAATCGGA	
pTac--*pfkA*-R	AAAACAGCCAAGCTTGGATCCCTACTCATCAAGGATCATAA	Construction of pHERD20T-based constructions for expression of MPAO1 KKP components
pHERD20T-*pfkB*-F	AGGAGATATACATACCCATGAGCACAATACCAGACCGATATGAATT	
pHERD20T-*pfkB*-R	ACGACGGCCAGTGCCAAGCTTTCATAAGTTTATCTCCGGATGAGATAG	
pHERD20T-*pfpC*-F	AGGAGATATACATACCCATGAATGTGAACTTAGAGAACGATATTACATTTC	
pHERD20T-*pfpC*-R	ACGACGGCCAGTGCCAAGCTTCTAGTCATCCTTGATGGGGAAAGA	
pfkA^{CSD}-R	ACGACGGCCAGTGCCAAGCTTTCAACATAAAAGTTCACATTTTTTTACC	Construction of pHERD20T-*pfkA*^{CSD}-*pfkB*, pHERD20T-*pfkA*-*pfkB*^{FHA} and
pfkB^{FHA}-R1	TTAGCGCTTGATGTCTGTTTTCTAACTATATGTGCTTCTCAAGTGTCTCTTTGATTGAC	
pfkB^{FHA}-F2	GTCAATCAAAGAGACACTTGAGAAGCACATATAGTTAGAAAACAGACATCAAGCGCTAA	

		pHERD20T-*pfkA*^{CSD}-*pfkB*^{FHA} together with primers pHERD20T-*pfkB*-F and pHERD20T-*pfkA*-R
pHERD20T-*pfkA*^{CSD}-R	ACGACGGCCAGTGCCAAGCTTTCATATGTGCTTCTCAAGTGTCTCTTT	Construction of pHERD20T-*pfkA*^{CSD}, pHERD20T-*pfkA*^{CSD}, pHERD20T-*pfkB*^{FHA} and pHERD20T-*FHA* together with primers pHERD20T-*pfkB*-F/R and pHERD20T-*pfkA*-F/R
pHERD20T-*CSD*-R	AGGAGATATACATACCCATGTTAGAAAACAGACATCAAGCGC	
pHERD20T-*pfkB*^{FHA}-R	ACGACGGCCAGTGCCAAGCTTCTAACATAAAAGTTCACATTTTTTTACCA	
pHERD20T-*FHA*-R	AGGAGATATACATACCCATGTATGCAAAAAACCCAAGATATATTG	
**Primers used for construction of conserved domains in pHERD20T-*pfkA-pfkB***		
pHERD20T-*pfkA*^{GGM}-*pfkB*-F	ATCTCGATATTTAATCGAGGACTTAATCGGCGAAGCAGCGTGTCAATATGTTTACCG	F primers/HERD20T-*pfkA*-R and pHERD20T-*pfkB*-F/R primers were used to amplify the second and first fragments separately, the two fragments were fused using pHERD20T-*pfkB*-F and pHERD20T-*pfkA*-R and ligated into pHERD20T plasmid.
pHERD20T-*pfkA*^{GGM}-*pfkB*-R	CGGTAAACATATTGACACGCTGCTTCGCCGATTAAGTCCTCGATTAAATATCGAGAT	
pHERD20T-*pfkA*^{HRD}-*pfkB*-F	TGCCGGAGTAGTTTTCGAAAATCTGAAACCAAC	
pHERD20T-*pfkA*^{HRD}-*pfkB*-R	GTTGGTTTCAGATTTTCGAAAACTACTCCGGCA	
pHERD20T-*pfkA*^{DFG}-*pfkB*-F	GTTAAAAATTACTAATTATGCAATTGCAAAAATG	
pHERD20T-*pfkA*^{DFG}-*pfkB*-R	CATTTTTGCAATTGCATAATTAGTAATTTTTAAC	
pHERD20T-*pfkA-pfkB*^{GGM}-F	TTGATGCAGCGTTAGGTACAATCTTCGTCTGC	
pHERD20T-*pfkA-pfkB*^{GGM}-R	GCAGACGAAGATTGTACCTAACGCTGCATCAA	
pHERD20T-*pfkA-pfkB*^{HRD}-F	GTAAACATAATTTTCGAAAATATCAAACCAAACAAC	
pHERD20T-*pfkA-pfkB*^{HRD}-R	GTTGTTTGGTTTGATATTTTCGAAAATTATGTTTAC	
pHERD20T-*pfkA-pfkB*^{DFG}-F	ATCATTAAAATATTTAATTATGCATTAGCAAGACACAC	
pHERD20T-*pfkA-pfkB*^{DFG}-R	GTGTGTCTTGCTAATGCATAATTAAATATTTTAATGAT	
pfkA-pfkB-linker-F	TTACGTTCGACCTATCTCATCCGGAGATAAACTTATGACGACTTCGAGAATCGGAAAGACTATA	Construction the double GGM, HDR, DFG mutations, the *pfkB* and *pfkA* single mutants were amplified using the above PCR products as templates, and fused with the primers and ligated into pHERD20T plasmid.
pfkA-pfkB -linker-R	TATAGTCTTTCCGATTCTCGAAGTCGTCATAAGTTTATCTCCGGATGAGATAGGTCGAACGTAA	
pfkB-in-R1	TAAGTCATTGCCGTAGATATATTCTTGTACGATGCCAAGCCCGCGTTCG	Construction of pHERD20T-*pfkA*$^{GGM+HRD+DFG}$ -*pfkB*, pHERD20T-*pfkA-pfkB*$^{GGM+HRD+DFG}$ and
pfkB-in-F1	CGAACGCGGGCTTGGCATCGTACAAGAATATATCTACGGCAATGACTTA	
pfkB-in-R2	AATATTTTAATGATGCTTTCATGGTCTAGCATCATGTTGTTTGGTTTGA	
pfkB-in-F2	TCAAACCAAACAACATGATGCTAGACCATGAAAGCATCATTAAAATATT	

pfkA-in-R1	GGCTACGTTATGATGATTGACTTTGGCTGCTACAATGGCACTACGCCG	pHERD20T-*pfkA*$^{GGM+HRD+DFG}$-*pfkB*$^{GGM+HRD+DFG}$, mutant *pfkB* and *pfkA* with were amplified using above mutants as templates, and fused with these primers, and *pfkA*-*pfkB*-linker-F/R, then ligated into pHERD20T plasmid.
pfkA-in-F1	CGGCGTAGTGCCATTGTAGCAGCCAAAGTCAATCATCATAACGTAGCC	
pfkA-in-R2	AACTCATTTAGTGAATATCCGCCAGAGATCATAACGTTTGTTGGTTTCA	
pfkA-in-F2	TGAAACCAACAAACGTTATGATCTCTGGCGGATATTCACTAAATGAGTT	

Primers used for mutation of potential active sites in pHERD20T-*pfkA*-*pfkB*

pfkA^{T34A}-*pfkB*-F	CTTTAATGCGACTTTTCTGCTAAGTAGAGCGTCATGGGCTCGGTAAACATA	
pfkA^{T34A}-*pfkB*-R	TATGTTTACCGAGCCCATGACGCTCTACTTAGCAGAAAAGTCGCATTAAAG	
pfkA^{T34D}-*pfkB*-F	CTTTAATGCGACTTTTCTGCTAAGTAGATCGTCATGGGCTCGGTAAACATA	
pfkA^{T34D}-*pfkB*-R	TATGTTTACCGAGCCCATGACGATCTACTTAGCAGAAAAGTCGCATTAAAG	
pfkA^{S37A}-*pfkB*-F	TTTAATGCGACTTTTCTGGCAAGTAGAGTGTCATGGGCTCG	
pfkA^{S37A}-*pfkB*-R	CGAGCCCATGACACTCTACTTGCCAGAAAAGTCGCATTAAA	
pfkA^{S37D}-*pfkB*-F	CTTTAATGCGACTTTTCTGTCAAGTAGAGTGTCATGGGCTCGG	
pfkA^{S37D}-*pfkB*-R	CCGAGCCCATGACACTCTACTTGACAGAAAAGTCGCATTAAAG	
pfkA^{S72A}-*pfkB*-F	CCTCTTTTATGTAGTCTAGTGCTTTGGCTACGTTATGATGATTG	Construction of pHERD20T-*pfkA*-*pfkB* based single site mutations in PfkA or PfkB (the red letters are the mutant nucleotide bases)
pfkA^{S72A}-*pfkB* -R	CAATCATCATAACGTAGCCAAAGCACTAGACTACATAAAAGAGG	
pfkA^{S72D}-*pfkB*-F	GATTATCATCCTCTTTTATGTAGTCTAGATCTTTGGCTACGTTATGATGATTGACTTTGG	
pfkA^{S72D}-*pfkB* -R	CCAAAGTCAATCATCATAACGTAGCCAAAGATCTAGACTACATAAAAGAGGATGATAATC	
pfkA-*pfkB*^{T20A}-F	TGCAGACGAAGATTGCACCCATGCCGCCATC	
pfkA-*pfkB*^{T20A} -R	GATGGCGGCATGGGTGCAATCTTCGTCTGCA	
pfkA-*pfkB*^{T20D}-F	TAAAATTCTATCATTGCAGACGAAGATATCACCCATGCCGCCATCAATTTTTTTATC	
pfkA-*pfkB*^{T20D} -R	GATAAAAAAATTGATGGCGGCATGGGTGATATCTTCGTCTGCAATGATAGAATTTTA	
pfkA-*pfkB*^{S328A}-F	CCTGATTACTTCTTTGTGCATTGCCGATTGCCAAGACG	
pfkA-*pfkB*^{S328A}-R	CGTCTTGGCAATCGGCAATGCACAAAGAAGTAATCAGG	
pfkA-*pfkB*^{S328D}-F	GCGCTCCTGATTACTTCTTTGATCATTGCCGATTGCCAAGACGCA	
pfkA-*pfkB*^{S328D}-R	TGCGTCTTGGCAATCGGCAATGATCAAAGAAGTAATCAGGAGCGC	

Primers used for constructing pMD19 based clones

pMD19-*pfiA*-F	ATGCGAGTCGAGACAATTAGTTATTTGAA
pMD19-*pfiA*-R	TTATTCTGGCTGAGCGAACCTCCTGGAAAG
pMD19-*intF6*-F	ATGACGGTACGCAAGGACGGCAAGACGTG

pMD19-*intF6*-R	CTACTGATCTACCTGACTAGCGAAGTGCT

Primers used for constructing clones for purification and pull-down assay

pET28b-*pfkA*-His-F	TAAGAAGGAGATATACCATGACGACTTCGAGAATCGGAAAGACTATA
pET28b-*pfkA*-His-R/ pET28b-*pfkB*$_{MP}$-*pfkA*$_{MP}$-His-R/ pET28b-*pfpC*$_{MP}$-*pfkB*$_{MP}$-*pfkA*$_{MP}$-His-R	CTCGAGTGCGGCCGCAAGCTTTTAGTGATGATGATGATGATGCTCATCAAGGATCATAACAGGATGTGCTCTCC
pET28b-*pfkB*-His-F/ pET28b-*pfkB*$_{MP}$-*pfkA*$_{MP}$-His-F	TAAGAAGGAGATATACCATGAGCACAATACCAGACCGATATGA
pET28b-*pfkB*-His-R	CTCGAGTGCGGCCGCAAGCTTTTAGTGATGATGATGATGATGTAAGTTTATCTCCGGATGAGATAGGTCG
pET28b-*pfpC*$_{MP}$-*pfkB*$_{MP}$-*pfkA*$_{MP}$-His-F	TAAGAAGGAGATATACCATGACGACTTCGAGAATCGGAAAGACTATA
pET28b-His-*pfkB*$_{MP}$-F	CTTTAAGAAGGAGATATACATGCATCATCATCATCATCACAGCACAATACCAG
pET28b-*pfkA*$_{MP-Flag}$-R	GTGCTCGAGTGCGGCCGCAAGCTTTTACTTATCGTCGTCATCCTTGTAATCCTCATCAAGGATCATAACAGGATGTGCTCTCC
pET28b-His-*pfkA*$_{MP}$(N)-F	TAAGAAGGAGATATACCATGCATCATCATCATCATCACACGACTTCGAGAATCGGAAAG
pET28b-His-*pfkA*$_{MP}$(N)-R	CTCGAGTGCGGCCGCAAGCTTCTAACATAAAAGTTCACATTTTTTTACCA
pET28b-His-*FHA*-F	TAAGAAGGAGATATACCATGCATCATCATCATCATCACTTAGAAAACAGACATCAAGCG
pET28b-His-*FHA*-R	CTCGAGTGCGGCCGCAAGCTTTCATAAGTTTATCTCCGGATGAGATAG
pET28b-F/pET21a-F/ pET28a-Tev-F	TAATACGACTCACTATAGGG
pET28b-R/pET21a-R/ pET28a-Tev-R	TATGCTAGTTATTGCTCAG
pET21-*pfkB*$_{MP}$(N)-Flag-F	AAGAAGGAGATATACATATGAGCACAATACCAGACCGATA
pET21-*pfkB*$_{MP}$(N)-Flag-R	TGGTGGTGGTGGTGCTCGAGCTACTTATCGTCGTCATCCTTGTAATCTATGTGCTTCTCAAG
pET21-*CSD*-Flag-F	AAGAAGGAGATATACATATGTATGCAAAAAACCCAAGATA
pET21-*CSD*-Flag-R	TGGTGGTGGTGGTGCTCGAGTCACTTATCGTCGTCATCCTTGTAATCCTCATCAAGGATCAT
pET28a-Tev-*gp59*-F/ pET28a-Tev-*gp59*^{G32E}-F	AAAACCTGTATTTTCAGGGCCATATGATTAAACTCCGCATGCC
pET28a-Tev-*gp59*-R/ pET28a-Tev-*gp59*^{G32E}-R	CGAGTGCGGCCGCAAGCTTTCA ATACTTGCAAGATTTCACAG

pETduet-His-*pfpC*$_{EC039}$-F	AAGAAGGAGATATACCAGG ATGCATCATCATCATCATCACAGCCGTGATTCTTACGAA	
pETduet-His-*pfpC*$_{EC039}$-R	AGCATTATGCGGCCGCAAGCTT TCACTCATCATCACCATCTCC	
pETduet-His-*gp59*-F/ pETduet-His-*gp59*^{G32E} -F	ATAAGAAGGAGATATACATATG ATTAAACTCCGCATGCC	
pETduet-His-*gp59*-R/ pETduet-His- *gp59*^{G32E}-R	CGGTTTCTTTACCAGACTCGAG ATGTCAATACTTGCAAGATTTCA	
pETduet-F1	ATGCGTCCGGCGTAGAGGAT	
pETduet-R1	GATTATGCGGCCGTGTACAA	
pETduet-F2	TTGTACACGGCCGCATAATC	
pETduet-R2	TGCTAGTTATTGCTCAGCGG	
**Primers used for cloning *Shewanella* W3-18-1 KKP$_{SW}$ components**		
pHERD20T-*2208*-F	AGGAGATATACATACCCATGTTTCAAAGGCTATTGCAAAAA	Construction of pHERD20T-based constructions for expression of KKP$_{SW}$ components and relative mutants
pHERD20T-*2208*-R	ACGACGGCCAGTGCCAAGCTTCTACTTGGAATCATCATCTTCAACCT	
pHERD20T-*2209*-F	AGGAGATATACATACCCATGATGATTCCAAGTAGATATGAACTCTG	
pHERD20T-*2209*-R	ACGACGGCCAGTGCCAAGCTTTCATGAAACCACCTCTGGGTTAG	
pHERD20T-*2210*-F	AGGAGATATACATACCCATGATCACATCGAATACTCTGATCG	
pHERD20T-*2210*-R	ACGACGGCCAGTGCCAAGCTTTTAATTTAAGATAATGACTGGGTGAGC	
pTac-KKP$_{SW}$-F	CAATTTCACACAGGAGAATTCATGTTTCAAAGGCTATTGCAAAAA	
pTac-KKP$_{SW}$-R	AAAACAGCCAAGCTTGGATCCTTAATTTAAGATAATGACTGGGTGAGC	
pTac-K^{GFA}KP$_{SW}$-F1	CAATTTCACACAGGAGAATTCATGTTTCAAAGGCTATTGCAAAAA	
pTac-K^{GFA}KP$_{SW}$-R1	ATTCAAAACGGATATAGTGACTATGCTTCAAACCCTCAACATAGC	
pTac-K^{GFA}KP$_{SW}$-F2	GCTATGTTGAGGGTTTGAAGCATAGTCACTATATCCGTTTTGAAT	
pTac-K^{GFA}KP$_{SW}$-R2	AAAACAGCCAAGCTTGGATCCTTAATTTAAGATAATGACTGGGTGAGC	
**Primers for cloning pTac based KKP$_{MP}$ and KKP$_{EC039}$ clones**		
pTac-*pfkA*-F	CAATTTCACACAGGAGAATTCATGACGACTTCGAGAATCGGA	Construciton of pTac-*pfkA*, pTac-*pfkB* and pTac- KK$_{MP}$
pTac-*pfkA*-R	AAAACAGCCAAGCTTGGATCCCTACTCATCAAGGATCATAA	
pTac-*pfkB*-F	CAATTTCACACAGGAGAATTCATGAGCACAATACCAGAC	
pTac-*pfkB*-R	AAAACAGCCAAGCTTGGATCCTCATAAGTTTATCTCCGGATGA	
pTac-*pfkB*$_{EC039}$-F	CAATTTCACACAGGAGAATTCATGAGTGATTTTCTCCCAGAAAGATA	Construciton of pTac-*pfkB*$_{EC039}$ and pTac- KKP$_{Ec039}$
pTac-*pfkB*$_{EC039}$-R	AAAACAGCCAAGCTTGGATCCTCATAACACAACCTCCGGATGA	
pTac- KKP$_{Ec039}$-F	CAATTTCACACAGGAGAATTCATGAGCCGTGATTCTTACGAAAT	

pTac- KKP$_{Ec039}$-R	AAAACAGCCAAGCTTGGATCCCTACTTAGCCTTGATAACTGGATGAGC	
**Plasmid verification primers**		
pEX18Ap-F/pEX18Gm-F	AATCTTCTCTCATCCGCCAAAACA	
pEX18Ap-R/pEX18Gm-R	CGCCCAATACGCAAACCGCCTCTC	
pHDRD20T-F	ATCGCAACTCTCTACTGTTTCT	Verification of constructions used for gene knockout and expression
pHDRD20T-R	TGCAAGGCGATTAAGTTGGGT	
pTac-F	CACCTCGCTAACGGATTCACC	
pTac-R	CCAATACGCAAACCGCCTC	
pMD19-F	TGTAAAACGACGGCCAGT	
pMD19-R	CAGGAAACAGCTATGACC	
**QPCR/QRT-PCR primers**		
attP-Pf4-F	GGGCTTGGCAGGGTGATT	
attP-Pf4-R	TCATGCAGGACCTGTCGAAA	
pfkA-qF	CGACCTGAAACCAACAAACGT	
pfkA-qR	TTAAACCATTGCTGAAAGGGAA	
pfkB-qF	GAAATGAGGTGCGTCGGTAT	
pfkB-qR	ACAAGAATATATCTACGGCAATGAC	
pfpC-qF	GCCATGACAACTGCTCCACT	
pfpC-qR	TTTTCTATGCTCGCTTGCTTT	
xisF4-qF	CCGCAACAGGATGTGGAG	
xisF4-qR	GCACTCCATTCCTGTTCCAA	
xisF6-qF	GCCTGGAAGACATCACGGAAGAC	
xisF6-qR	CCTGGCAGAAGATCGACTTACCC	
repF-qF	GGCGTGCTGGATGATTTGG	
repF-qR	GACAGTTGCAGGCCGTTGG	
intF6-qF	GCTACTTGTCGGCGGTCTTC	
intF6-qR	GATTCTTGGTCCGGTGATAGTG	
16S rRNA-qF	TGGTTCAGCAAGTTGGATGTG	
16S rRNA-qR	GTTTGCTCCCCACGCTTTC	
gyrB-qF	CAAGTACGAAGGCGGTCTGAAG	
gyrB-qR	GCAGAGCAGGTTCTCGTTGAA	

Supplementary References

1 Chandler, C. E. *et al.* Genomic and phenotypic diversity among ten laboratory isolates of *Pseudomonas aeruginosa* PAO1. *J. Bacteriol.* **201**, e00595-00518 (2019).

2 Burkinshaw, B. J. *et al.* A type VI secretion system effector delivery mechanism dependent on PAAR and a chaperone-co-chaperone complex. *Nat. Microbiol.* **3**, 632-640 (2018).

3 Li, Y. *et al.* Excisionase in Pf filamentous prophage controls lysis-lysogeny decision-making in *Pseudomonas aeruginosa*. *Mol. Microbiol.* **111**, 495-513 (2019).

4 Hoang, T. T., Karkhoff-Schweizer, R. R., Kutchma, A. J. & Schweizer, H. P. A broad-host-range Flp-*FRT* recombination system for site-specific excision of chromosomally-located DNA sequences: application for isolation of unmarked *Pseudomonas aeruginosa* mutants. *Gene* **212**, 77-86 (1998).

5 Qiu, D. R., Damron, F. H., Mima, T., Schweizer, H. P. & Yu, H. D. P-BAD-based shuttle vectors for functional analysis of toxic and highly regulated genes in *Pseudomonas* and *Burkholderia* spp. and other bacteria. *Appl. Environ. Microbiol.* **74**, 7422-7426 (2008).

6 Wang, W. *et al.* Filamentous prophage capsid proteins contribute to superinfection exclusion and phage defence in *Pseudomonas aeruginosa*. *Environ. Microbiol.* **24**, 4285–4298 (2022).

7 Li, Y. *et al.* Prophage encoding toxin/antitoxin system PfiT/PfiA inhibits Pf4 production in *Pseudomonas aeruginosa*. *Microb. Biotechnol.* **13**, 1132-1144 (2020).
