# Supplementary material for: Control of lysogeny and antiphage defense by a prophage-encoded kinase-phosphatase module
Source: Nat Commun. 2024 Aug 23;15:7244. doi: 10.1038/s41467-024-51617-x (PMC11341870; doi:10.1038/s41467-024-51617-x)
Supplement: Supplementary file 3 — Description of Additional Supplementary Files [file 41467_2024_51617_MOESM3_ESM.pdf]

Description of Additional Supplementary Files:

Supplementary Data 1: Published articles with sequencing raw data deposited in the NCBI SRA database. Sequencing reads were mapped to the reference genome of MPAO1 (CP079712). Average depths were calculated of Pf4 and Pf6 genome regions as well as the upstream and downstream 1000 bp regions flanking the prophage genomes. Y indicates yes and N indicates no.

Supplementary Data 2: Distribution of KKP modules in bacteria. Data were retrieved from the IMG/G database. Gene IDs are indicated.

Supplementary Data 3: Genomes used for KKP searching.

Supplementary Data 4: List of phosphorylation sites identified by phosphoproteomics.
